# Phosphorus Chemistry and Bacterial Community Composition Interact in Brackish Sediments Receiving Agricultural Discharges

**DOI:** 10.1371/journal.pone.0021555

**Published:** 2011-06-29

**Authors:** Hanna Sinkko, Kaarina Lukkari, Abdullahi S. Jama, Leila M. Sihvonen, Kaarina Sivonen, Mirja Leivuori, Matias Rantanen, Lars Paulin, Christina Lyra

**Affiliations:** 1 Department of Food and Environmental Sciences, University of Helsinki, Helsinki, Finland; 2 Marine Research Centre, Finnish Environment Institute, Helsinki, Finland; 3 National Institute for Health and Welfare, Helsinki, Finland; 4 Reference Laboratory, Finnish Environment Institute, Helsinki, Finland; 5 Institute of Biotechnology, University of Helsinki, Helsinki, Finland; Queen Mary University of London, United Kingdom

## Abstract

**Background:**

External nutrient discharges have caused eutrophication in many estuaries and coastal seas such as the Baltic Sea. The sedimented nutrients can affect bacterial communities which, in turn, are widely believed to contribute to release of nutrients such as phosphorus from the sediment.

**Methods:**

We investigated relationships between bacterial communities and chemical forms of phosphorus as well as elements involved in its cycling in brackish sediments using up-to-date multivariate statistical methods. Bacterial community composition was determined by terminal restriction fragment length polymorphism and cloning of the 16S rRNA gene.

**Results and Conclusions:**

The bacterial community composition differed along gradients of nutrients, especially of different phosphorus forms, from the estuary receiving agricultural phosphorus loading to the open sea. This suggests that the chemical composition of sediment phosphorus, which has been affected by riverine phosphorus loading, influenced on bacterial communities. Chemical and spatial parameters explained 25% and 11% of the variation in bacterial communities. *Deltaproteobacteria*, presumptively sulphate and sulphur/iron reducing, were strongly associated to chemical parameters, also when spatial autocorrelation was taken into account. Sulphate reducers correlated positively with labile organic phosphorus and total nitrogen in the open sea sediments. Sulphur/iron reducers and sulphate reducers linked to iron reduction correlated positively with aluminium- and iron-bound phosphorus, and total iron in the estuary. The sulphate and sulphur/iron reducing bacteria can thus have an important role both in the mineralization and mobilization of nutrients from sediment.

**Significance:**

Novelty in our study is that relationships between bacterial community composition and different phosphorus forms, instead of total phosphorus, were investigated. Total phosphorus does not necessarily bring out interactions between bacteria and phosphorus chemistry since proportions of easily usable mobile (reactive) phosphorus and immobile phosphorus forms in different sediments can vary. Our study suggested possible feedbacks between different forms of phosphorus and bacterial community composition.

## Introduction

Eutrophication is an expanding environmental problem in many coastal marine ecosystems [1]. One of the world's largest brackish water environments, the Baltic Sea, is severely eutrophied due to high levels of anthropogenic nutrient loading such as agricultural nutrient inputs. Large scale nutrient deposition has increased anoxic areas and hydrogen sulphide concentrations enhancing phosphorus release from bottom sediment [2], [3]. Recently, the role of phosphorus in marine eutrophication has been emphasized worldwide [4], [1], [3]. Phosphorus accelerates the growth of primary producers such as cyanobacteria and eukaryotic algae. Degradation of organic matter derived from cyanobacteria and algal blooms further increases anoxia in bottom waters in the stratified Baltic Sea and thus the release of phosphorus from sediments to the water column [5], [3]. Phosphorus can be released either from inorganic or organic compounds. Redox-sensitive, iron-bound phosphorus is released as inorganic phosphate when iron (Fe) oxyhydroxides, which bind phosphorus, are reduced under anoxic conditions [6], [7]. Degradation of organic phosphorus compounds release dissolved phosphate and smaller organic P compounds [8]–[9].

The effects of iron-bound phosphorus on sediment phosphorus release are well-known phenomena in the Baltic Sea [10], whereas the fate of organic phosphorus has not been widely studied. Recently, sequential extraction was used to investigate the abundance and behaviour of organic and inorganic phosphorus forms in sediments of the north-eastern Baltic Sea [11]–[13]. Organic phosphorus constitute a notable part of the phosphorus deposited in Baltic Sea sediments and thus can be an important source of continuous phosphorus release [10]–[13].

Prokaryotes participate in biogeochemical processes, such as phosphorus cycling in sediment [14]. Bioavailability of different organic and inorganic phosphorus forms varies with environment. Certain bacteria are able to utilize even recalcitrant phosphorus forms [15], [16]. Therefore, bacteria can play an important role in the release of sediment phosphorus. The microbe-mediated release of phosphorus is a black box in the brackish Baltic Sea sediment. Besides, other associations between sediment bacterial communities and environment in the Baltic Sea have been only little studied. Edlund *et al.* [17], [18] reported a change in bacterial community composition in Baltic Sea sediments along a horizontal coastal pollution gradient and vertical redox-gradients. To understand how eutrophication affects sediment bacterial communities and how nutrients, particularly phosphorus, are withdrawn from the biosphere or released into it, a combined investigation on bacterial communities and chemical parameters from horizontal and vertical ranges of sediments is needed.

In Finland, agriculture accounts for most of the anthropogenic phosphorus load to the water bodies (lakes, bonds, rivers etc.). The Baltic Sea receives the majority of these loads [19], [20]. Especially the Archipelago Sea is high in area-specific phosphorus load from agricultural origin [21]. The steep riverbanks of the agriculture-intensive drainage area in south-western Finland are prone to erosion as a consequence of crop cultivation on clayey soils [19], [22]. This results in high loads of suspended solids [23], rich in particulate phosphorus [22], [24], and dissolved phosphorus [23] to the Archipelago Sea. In addition to the quality and amount of riverine phosphorus loading, sedimentation environment affects to the chemical forms of phosphorus occurring in recipient sediments. Different forms of phosphorus, in turn, vary with their bioavailability which impacts bacterial community composition.

We hypothesized that the occurrence of sediment phosphorus in various forms, partly as a result of riverine phosphorus loading from the drainage area in south-western Finland, affects bacterial community composition, which cause feedbacks between bacterial communities and sediment chemistry. To find support for this hypothesis, we investigated changes in bacterial communities relative to trends in chemical phosphorus forms, as well as elements participating in phosphorus binding in brackish sediments. In addition, we identified which bacteria correlated with different forms of phosphorus and thus may play a role in phosphorus release. The bacterial communities were identified, using molecular methods in sediment samples from horizontal and vertical scales of the north-eastern Baltic Sea bottom area. The bacterial data, produced in this study, and phosphorus forms [11]–[13] from the same samples were analysed with up-to-date multivariate statistics. The novelty in our study is that we examine changes in bacterial communities along gradients of different phosphorus forms in brackish sediments, although biased causality in correlations between bacterial and phosphorus data by other environmental factors could not completely ruled out. Phosphorus represented mobile and immobile organic and inorganic phosphorus forms (see detailed description in Table 1). Such an interdisciplinary study has not been implemented to date to our knowledge. The bacterial communities varied from the estuary to the open sea along a gradient of different phosphorus forms. Iron/sulphur reducing bacterial taxa were common in estuary sediments rich in iron-bound phosphorus whereas sulphate reducing taxa were abundant in the open sea rich in labile organic phosphorus.

**Table 1:** Phosphorus forms, their potential biodegradability in sediment and their potential environmental effects.

Used definition of phosphorus forms^a^	Classification of phosphorus forms	Examples of phosphorus compounds^e^	Potential biodegradability or bioavailability^i^	Potential environmental effect
Pore water P, loosely adsorbed P^b^	Dissolved inorganic P	Phosphate (PO_4_-P)	Already biodegraded or released from sorption sites	Increases eutrophication
Iron-bound P, redox-sensitive P b	P bound to hydrated oxides of reducible metals, mainly those of Fe	Phosphate (PO_4_-P) bound to hydrated oxides of Fe^3+^	Biodegradable or released if Fe-compounds are reduced	Increases eutrophication
Labile organic P^b^	Low molecular weight dissolved organic P^c^	Orthophosphate monoesters^f^ and diesters^g^, poly-P compounds^h^	Partly biodegradable (includes also degradation products)	Increases eutrophication
Refractory organic P^b^	High molecular weight particulate organic P^d^	E.g. phosphonates	Slowly biodegradable^j^, mainly recalcitrant	Mainly buried with sediment in shallow seas, decreases eutrophication
Aluminium-bound P^b^	P bound to hydrated oxides of non-reducible metals, mainly those of Al	Phosphate (PO_4_-P) bound to hydrated oxides of Al^3+^	Mainly unavailable, bioavailable only if released from Al-compounds	Buried with sediment, decreases eutrophication
Apatite P^b^	P in apatite minerals	Detrital apatite minerals, may include authigenic apatite	Mainly unavailable, may be slowly biodegradable^k^	Buried with sediment, decreases eutrophication if includes authigenic apatite-P forms

a See phosphorus fractionation method in Table S2.

b In this fractionation method, according to a coarse division, pore water and loosely adsorbed P, redox-sensitive (iron-bound) P, and labile organic P are considered mobile (or reactive) phosphorus forms while refractory P, aluminium-bound P, and apatite-P are considered immobile phosphorus forms [25], [11].

c Particle size <0.4 µm.

d Particle size >0.4 µm.

e [26], [25].

f e.g. sugar phosphates, mononucleotides, phospholipids, inositol P.

g e.g. sugar DNA-P, lipid P, teichoic-P.

h e.g. adenosine triphosphate.

i Biodegraded to or chemically released phosphorus which is bioavailable.

j [27]–[29].

k [30], [16].

## Materials and Methods

### Research area and sediment properties

**Figure 1:**
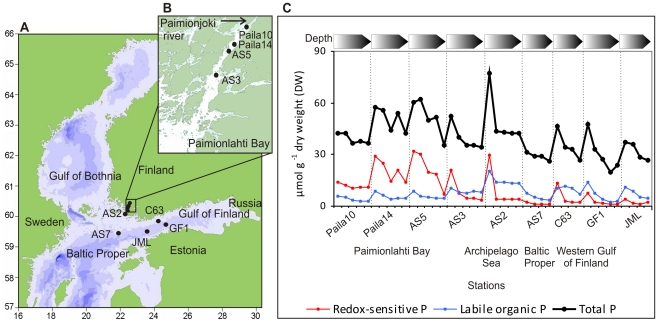
The research area and the sediment sampling stations. (A) The sampling stations in Paimionlahti Bay (Paila10, Paila14, AS5, and AS3), in the Archipelago Sea (AS2), in the northern Baltic Proper (AS7), and in the western Gulf of Finland (JML, GF1, and C63). (B) A magnification of Paimionlahti Bay and the sampling sites located in the estuary. (C) Concentrations of reactive phosphorus (P) forms; redox-sensitive and labile organic P, and total P in the solid phase of the sediment samples. Arrows (in the upper part of the panel C) indicate sediment depth from 1 cm down to 25 cm below the seafloor (Refer to Dataset S2). Darkening colour of the arrows denotes the increasing subsurface depth.

Sediment samples were collected from the Paimionjoki River estuary (Paimionlahti Bay, referred here as Paila), from the coastal sites (Archipelago Sea and western Gulf of Finland) and from the open sea (Baltic Proper and western Gulf of Finland) (Figure 1A and B, Table S1). Sampling sites Paila10, Paila14, AS5 and AS3 from the estuary and AS2 from the Archipelago Sea, as well as AS7 from the Baltic Proper, formed a transect along a phosphorus gradient. The concentration of total phosphorus in the sediment was highest in Paimionlahti Bay and the Archipelago Sea (Figure 1C, Dataset2) due to nutrient loadings from the Paimionjoki River [21], which flows through the agriculture-intensive drainage area of the southwestern Finland. The loads of total phosphorus and nitrogen as well as phosphate in 1995 from the Paimionjoki River were 60, 819 and 37.7 t^−1^, respectively [21]. The amount of total phosphorus loading was high compared to most of other Finnish riverine loadings [21]. No significant reduction in nutrient loading was detected by year 2003 [23] when the sampling for this study took place.

The chemical character of sediment phosphorus varied along the transect (Dataset S2) [11]–[13]. Briefly, redox-sensitive iron-bound phosphorus was abundant in the surface sediment of Paimionlahti Bay [11], whereas iron-bound and labile organic phosphorus were abundant in the Archipelago Sea and organic and apatite phosphorus in the western Gulf of Finland (Figure 1C) [12], [13]. In deeper sediments, immobile aluminium-bound (alkali-extractable inorganic) and iron-bound phosphorus dominated in the estuary area [11] and apatite phosphorus (HCl-extractable) in the open sea area [12], [13]. The oxygen concentration of the near-bottom (hypolimnic) water was below 2 ml l^−1^ at sampling sites AS5, AS7, JML and GF1. At other sampling sites the near-bottom water was oxic (O_2_>2 ml l^−1^) and there were signs of bioturbation in the sediment. The oxygen concentrations and important sediment properties such as incubation-derived phosphate (PO_4_-P) flux from sediment to the water column are summarized in Table S1. The incubation-derived phosphate flux varied highly among the sites but it was highest at the anoxic site GF1. Pore water and near-bottom water phosphate concentrations increased from the estuary area towards the open sea indicating phosphorus release from hypoxic sediments [11]–[13]. Further details on the research area are presented in Lukkari *et al*. [11]–[13].

### Sediment sampling

The samples were collected with a Gemax gravity corer (two acrylic cylinders, inner diameter 9 cm, length app. 60 cm) from nine sampling sites (Figure 1A and B) during two cruises on the r/v Aranda (assisted by the r/v Aurelia) in September 2003 and in April 2004 (site C63). Two parallel sediment cores were cut into 1-cm slices from depths 0–1, 6–7, 14–15, 19–20 and 24–25 cm, except the core from site C63, which was cut from depths 0–1, 1–2, 4–5 and 9–10 cm. The corresponding depth layers were pooled and homogenized. Of this sample, three parallel 2-ml subsamples were collected for microbiological analyses and stored at −20°C during the cruise and at −70°C after the cruise. All the handling steps from the core sectioning to sample storing were performed under a nitrogen atmosphere (O_2_ content <5–10%). Further details on sediment sampling can be found in [11] (stations Paila10, Paila14, AS5, and AS3), in [12] (stations C63 and AS2) and in [13] (stations AS7, JML, and GF1).

### Chemical data

Different chemical forms of sediment phosphorus and elements participating in phosphorus binding (or related to it) (Fe, Mn, Al, Ca, Mg, Si) from phosphorus extraction solutions, as well as total concentrations of elements (P, N, C, S, Fe, Mn, Al, Ca) from the sediment solid phase were analysed previously [11]–[13]. Of all chemical data, the parameters that were used in the final statistical analyses are shown in Dataset S2. Analysis of chemical forms of phosphorus, using a slightly modified fractionation method by Jensen and Thamdrup [31], is described in detail by Lukkari *et al*. [25], [32] and summarized in Table S2. Summary of phosphorus forms, their reactivity, biodegradability or bioavailability in sediments, and their potential environmental effects are presented in Table 1.

### DNA extraction and amplification of the 16S rRNA gene

The sediment samples were homogenized and DNA was extracted from approximately 0.3 g of each sediment sample (pore water removed by 30-s centrifugation at 10 000 g) using a Power Soil DNA extraction kit (MoBio Laboratories, Inc., Carlsbad, CA, USA). The DNA was used as a template in triplicate 16S rRNA gene amplifications for terminal restriction fragment length polymorphism (T-RFLP) analysis [33] and for constructing 16S rRNA clone libraries. The primers FAM27f, labelled at the 5′ terminus with 6-carboxyfluorescein (FAM-gagtttgatcmtggctcag) [34] (Oligomer Oy, Helsinki, Finland) and 1405r (acgggcggtgtgta) (Oligomer Oy), modified from 1406r [35] were used in a Polymerase chain reaction (PCR) for T-RFLP. In a PCR for cloning, primer 27f without label was used instead. Each PCR reaction for T-RFLP and for cloning was performed in a total volume of 100 µl and 25 µl of 1x reaction buffer (Finnzymes Oy, Espoo, Finland), respectively. The reaction buffer contained 0.2 µM of both primers, 0.2 mM of each deoxynucleoside triphosphate (Finnzymes Oy), 0.15 mM MgCl_2_ (Finnzymes Oy), 2U of DyNAzyme™ II DNA polymerase (Finnzymes Oy), and approximately 10–20 ng of DNA in reaction for T-RFLP and 7 ng in reaction for cloning. The amplifications were performed in an iCycler (Bio-Rad Laboratories, Hercules, CA, USA) with an initial denaturing step of 3 min at 94°C, followed by 30 cycles of 30 s of denaturing at 94°C, 30 s of annealing at 52°C, 60 s of extension at 72°C, and final extension of 10 min at 72°C. Three amplification products for both T-RFLP and cloning were pooled and purified, using an EZNA Cycle Pure kit (Omega Bio-Tek Inc., Norcross, GA, USA). If non-specific amplification products were formed, amplicons were purified from 1% agarose gel using MinElute™ Gel extraction kit (Qiagen, Hilden Germany).

### Terminal restriction fragment length polymorphism analysis

In all, 200 ng of purified and labelled amplification products were digested separately with 5 U of the HaeIII, HhaI, MspI and RsaI restriction enzymes (Promega Corp., Madison, WI, USA) in reaction buffer C for 2 h at 37°C. Four different enzymes were used to increase accuracy of terminal restriction fragment (TRF) data to reflect the natural diversity of microbial populations since one TRF can represent more than one bacterium [36], [37]. The separate restriction digestion products (1 µl) were mixed with 20 µl of HiDi formamide (Applied Biosystems, Foster City,CA, USA) and 0.2 µl of MapMapper® 1000 (Bioventures inc., Murfreesboro, TN, USA) internal size standard. The digestions were denatured at 95°C for 5 min. The fluorescently labelled T-RFs were separated by size on a 3130×l Genetic Analyzer (Applied Biosystems) with electrophoresis at 60°C with 15 kV for 30 min using the polymer POP7. The sizes of the T-RFs were determined with MapMarker® 1000 internal size standard (Bioventures) with Peak Scanner software (Applied Biosystems), using Local Southern method. The background noise was removed, based on normalized T-RFs separately for each dataset (produced by different restriction endonucleases) by IBEST tools [38] written in Perl and R languages. All T-RFs over baseline fluorescence units and with lengths between 50 and 700 base pairs (bp) were included in normalization. Five-fold standard deviation instead of the default option (three-fold) was used as a threshold for distinguishing background ‘noise’ from ‘true’ signal peaks since the signal peaks with notably high fluorescence intensity were rarely present in the raw data. In other words, most of the T-RFs representing bacterial species/taxa were roughly evenly abundant and highly dominant or minor species were rare. Thus, five-fold standard deviation did not favour highly abundant species at the expense of low abundant ones but ensured that background ‘noise’ was properly removed. True peaks of comparable size from different samples were binned to compensate for analytical errors in fragment-size determination [38]. The resulting data matrix for each T-RF data set (HaeIII, HhaI, MspI and RsaI) with relative abundance of each binned fragment (Dataset S1) was used in statistical analyses.

### 16S rRNA gene clone libraries and sequence analysis

The 16S rRNA gene was cloned, using a TOPO TA Cloning® kit (Invitrogen, Carlsbad, CA, USA), the PCR® 2.1.-TOPO® vector, and One Shot® TOP10F' Chemically Competent cells, according to the manufacturer's instructions. A colony-PCR was performed on randomly selected clones in a total volume of 30 µl of 1x reaction buffer containing 0.5 µM of M13f and M13r primers (Sigma-Aldrich, St. Louis, MO, USA), 50 µM of each dNTP (Finnzymes Oy), 0.15 mM MgCl_2_ (Finnzymes Oy) and DyNAzyme™ II DNA polymerase 1.2 U (Finnzymes Oy). The amplifications were performed with an iCycler (Bio-Rad laboratories) with an initial denaturing step of 10 min at 94°C, followed by 35 cycles of 30 s of denaturing at 94°C, 30 s of annealing at 56°C, 30 s of extension at 72°C and final extension of 10 min at 72°C. Approximately 500 bp from the 5′ terminus of the 16S rRNA genes were sequenced from the PCR products, using BigDye terminator chemistry, and analysed on an ABI 3130XL Genetic Analyzer. The 16S rRNA genes were taxonomically assigned, using a naive Bayesian classifier (version 2.2, RDP training set 6) of Ribosomal Database Project (RDP) [39]. The closest sequence matches were obtained from the RDP database (Release 10.10) using the seqmatch tool, version 3 [40], [41] of RDP. The search was performed against a dataset of both nontype and type strains, both near-full-length sequences (≥1200 bases) and short partials, using nomenclatural taxonomy and 20 matches per sequence. The 16S rRNA gene sequences have been assigned to the accession numbers from FN423817 to FN424050 in EMBL Nucleotide Sequence Database.

### Identification of T-RFs

The T-RFs were assigned to taxon by *in silico* (virtually) and *in vitro* digested 16S rRNA gene clones. The 16S rRNA gene clones were amplified as above (colony-PCR) and purified, using Millipore MultiSreen PCR 96 Kit. The purified amplification products were used as a template in amplification for T-RFLP using the primers FAM27f and 1405r described as above. 100 ng of the purified labelled amplification products were digested separately with 2.5 U of the restriction enzymes HaeIII, HhaI, and RsaI, in reaction buffer C and MspI in reaction buffer B for 2 h at 37°C. The fluorescently labelled T-RFs were separated by size on a 3130×l Genetic Analyzer (Applied Biosystems) as described above. The T-RFs were identified only if at least three different comparable T-RFs digested *in vitro* were found.

The virtual (*in silico*) taxon assignment for the T-RFs was based on virtually digested and assigned 16S rRNA gene clone sequences using the Bioperl tool T-DistinctiEnz hosted by the Bioinformatics Organization (http://www.bioinformatics.org/~docreza/rest_html/home.htm).

### Statistical analyses

The molecular microbiological, chemical and environmental parameters were analysed by the nonparametric distance-based multivariate methods [42]–[44]. These methods are suitable for T-RF data, such as ours, which include a high number of zeroes, are not normally distributed and where the number of variables (T-RFs) exceeds the number of observations. To decrease the number of variables, the appropriate distance-based principal coordinates, used in subsequent analysis, were first calculated. P values were calculated by permutations.

A subset of all 48 chemical parameters (n = 18) was selected to canonical analysis of principal coordinates (CAP) [42] using marginal tests of distance-based multivariate multiple regression (DISTML*forward* program) [43], [45]. In marginal tests each variable were fitted individually. The highly significant variables (P<0.01) where chosen to a preliminary CAP (Figure S1A) and partial CAP analysis (Figure S1B). CAP was performed in the R environment [46] using the package Vegan [47] (function “capscale” for CAP and function “permutest” for testing significance) to determine the relationships between bacterial T-RFs and chemical parameters as well as between individual bacterial communities and chemical parameters. In CAP, principal coordinates derived from T-RFs and chemical parameters were analysed by canonical correlation analysis. Partial CAP (Package Vegan [47], function “capscale” for partial CAP and function “permutest” for testing significance) was done to remove spatial autocorrelation, i.e. correlation of the chemical and bacterial parameters with geographic location (latitude and longitude) and sediment depth.

Based on comparison of the preliminary CAP and partial CAP (Figure S1), the variables which were not highly spatially dependent were tested using Akaike's information criterion (package Vegan [47], function “step”) as the selection criterion to find the final CAP model. The final chemical parameters (Dataset S2, excluding highly collinear and spatially dependent total carbon, total and HCl-extractable phosphorus) were also tested with distance-based multivariate multiple regression analysis with forward selection (conditional tests) (DISTML*forward* program) [43], [45]. The forward selection procedure determined those chemical variables that explained most of the variation in bacterial community composition.

Variance partitioning was done based on partial redundancy analysis (RDA). Series of distance-based RDA [43], [44] runs, performed according to Anderson and Gribble [48] using program DISTML [49], was subsequently used in partitioning the variation to pure chemical, environmental and spatial, as well as shared proportions [50]–[51], [48]. Detailed information of RDA runs is presented in supplementary materials (Table S3).

The Bray-Curtis distances were calculated between observations and 9999 permutations were used in all statistical analyses.

## Results

### Spatial structure of bacterial community composition

**Figure 2:**
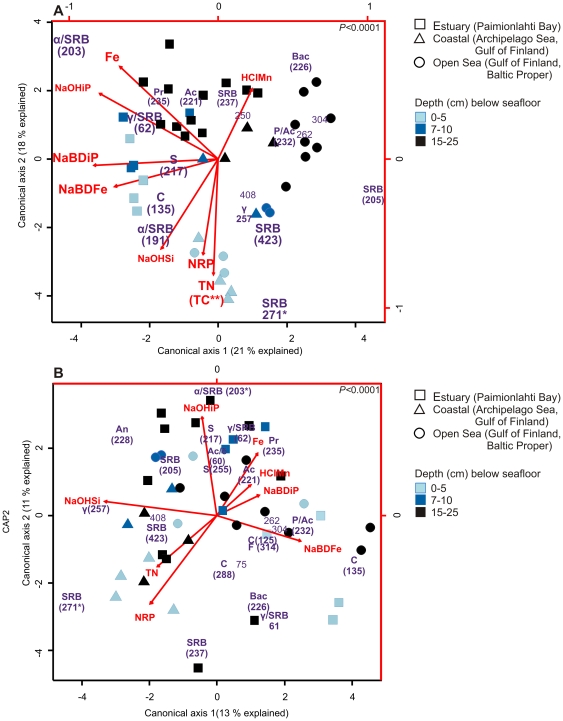
Relationships between bacterial community composition and chemical parameters of Baltic Sea sediments. (A) Canonical analysis of principal coordinates (CAP) and (B) partial CAP (spatial autocorrelation was excluded). Samples (n = 42) and HaeIII digested terminal restriction fragments (T-RFs, n = 104) and chemical parameters (red arrows, n = 8) were plotted against canonical axis scores 1 and 2. Black axes correspond to scores of samples/T-RFs and red axes to scores of chemical parameters. T-RFs and their corresponding taxonomic assignments are indicated with violet numbers (in bp) and letters. Large font size (A) shows those T-RFs which correlated to important chemical parameters. T-RFs of 16S rRNA gene were identified by digestion of cloned 16S rRNA genes (refer numbers to Table 2 and Table S4). Taxonomic assignments of TFs: Ac = *Actinobacteria*, α = *Alphaproteobacteria*, An = *Anaerolineae*, Bac =  *Bacteroidetes*, c = *Cyanobacteria*, δ = *Deltaproteobacteria*, F = *Firmicutes*, γ = *Gammaproteobacteria*, P = *Planctomycetasia*, Pr  = *proteobacteria*, SRB =  Sulphate reducers (*Deltaproteobacteria*), S = Sulphur/iron reducers (*Deltaproteobacteria*). Only T-RFs with canonical scores above ±1 for axis 1 and 2 were included. Scores were derived from canonical correlations. The arrow length indicates the strength of the correlation between the chemical parameter and sediment samples/T-RFs. The direction of an arrow indicates the increasing concentration of the chemical parameter. Chemical parameters: NaBDiP  =  iron-bound (redox-sensitive) phosphorus (P), NaBDFe  =  redox-sensitive iron (Fe), NaOHiP  =  aluminium-bound (alkali-extractable) P, NaOHSi  =  alkali-extractable silicon (Si), NRP  =  labile organic P, and HClMn  =  HCl-extractable manganese (Mn). TN  =  Total nitrogen (N), TC  =  Total carbon (C). *Positions of T-RFs 271 and 203 were changed for technical reasons. The real scores of T-RF 271 for axis 1 and 2 were 1.5 and −6.9 (A), and −8.8 and −1.5 (B). The axis scores T-RF 203 were −0.06 and 6.2 (B). ** Canonical scores of total carbon were nearly same than total nitrogen.

A clear spatial structure was seen in canonical analysis of principal coordinates (CAP), which determined individual associations between bacterial community composition and chemical data (Figure 2A). The bacterial community composition of sediments differed horizontally from the estuary to the open sea (Figure 2A, from left to right) and vertically from the surface to deep layers (Figure 2A, from down to up) along the gradient of different phosphorus forms and elements involved in its cycling (see description of phosphorus gradients in material and methods). In addition, elevated concentrations of total nitrogen and carbon, which were mostly organic, were associated to coastal and open sea bacterial communities in surface sediments. Sulphate and sulphur/iron reducers, which belong to class *Deltaproteobacteria,* were abundant from the surface sediment down to 15–25 cm (Figure 2A). CAP, which was based on HaeIII digested T-RFs, explained 57% of the variation in bacterial communities. CAP analyses based on T-RFs produced by HhaI, MspI and RsaI resulted in similar ordinations. (Figure S2A, S2C and S2E).

### Possible feedback interactions between bacterial communities and chemical parameters, with emphasis on phosphorus

In addition to changes in bacterial communities, the CAP analysis showed correlations between individual bacterial T-RFs and chemical parameters (Figure 2A). Above all, sulphate reducing bacteria (T-RFs 271,423, 191, Table 2) correlated positively with labile organic phosphorus, total nitrogen and alkali-extractable silicon. Sulphate reducing bacteria (T-RF 62, 203, Table 2), of which the latter (203) is linked to iron reduction, and *Proteobacteria* (T-RF 235, Table S4) associated with elevated concentrations aluminium-bound phosphorus and total iron. Potential sulphur/ferric iron reducer (T-RFs 217, Table 2) correlated positively with redox-sensitive iron and phosphorus. *Cyanobacteria*, presumably *Synechochoccus* (T-RF 135, Table S4), were associated with redox-sensitive iron (Figure 2A).

**Table 2:** Identification of 16S rRNA gene terminal restriction fragments of class *Deltaproteobacteria* from Baltic Sea sediments.

T-RF size (bp) a with													
	HaeIII			HhaI			MspI			RsaI			
clone	expected^b^	observed^c^	observedd,e	expected^b^	observed^c^	observedd,e	expected^b^	observed^c^	observedd,e	expected^b^	observed^c^	observedd,e	lowest rank^f^
JML-43	38	nd	29	92	88/90	88	504	503/505/506	504	487	487/488/490	488	*Bacteriovorax* (g)
JML-48	67	62/61	61	96	90/92	91	165	161/162	161	494	495	494	*Desulfobacterium* (g)
JML-70	71	71	65	83	76	78	200	201	199	441	440	440	*Deltaproteobacteria* (c)
Paila10-32	145	141	143	95	88/90/92	90	127	122	123	nd	490/492	491	*Desulfomonile* (g)
JML-67	178	nd	176	92	88/90	88	140	140	139	83	78/91	79	*Deltaproteobacteria* (c)
JML-37	183	nd	180	96	92	92	134	129/130	131/133^g^	57	52	50	*Desulfobacteraceae* (f)
JML-2	192	191/193	191/192^h^	94	88/90/92	90	163	162	163	213	211/213	212	*Desulfobacterales* (o)
JML-55	200	201/203	202	92	90/92	91	504	506/508	508	487	490/492	491	*Desulfovibrio* (g)
Paila10-52	204	203/205	203	94	88/90/92	90	143	nd	143	nd	490/492	492	*Deltaproteobacteria* (c)
JML-22	204	205/206	205	92	88/90/92	90	nd	510/511/512	512	225	226/227	226	*Desulfobulbaceae* (f)
JML-64	207	205/206	204	231	227	227	138	136/137	137	81	77/78	77	*Desulfobacterales* (o)
JML-5	208	205/206	205	96	88/90/92	90	69	62	62/64^g^	247	242/243	243	*Desulfobacteraceae* (f)
JML-29	208	205/206	206	379	378	378	165	165	164	57	52	51	*Desulfobacteraceae* (f)
Paila10-17	215	214/216	214	92	88/90	89	130	126/127/128	127	57	52	51	*Desulfuromonadaceae*(f)
Paila10-65	217	216/217	216	94	90/92	91	132	128/129/130	129	57	52/54	52	*Desulfuromonadales*(o)
JML-7	239	237/238	238	95	90/92	91	164	160/161/162	161	246	245/246	245	*Desulfobacteraceae* (f)
GF1-6	239	237/238	238	96	90/92	92	164	160/161/	161	246	245/246	245	*Desulfobacterium* (g)
Paila10-20	240	235/237	236	94	88/90/92	90	209	206/209	208	nd	290	289	*Deltaproteobacteria* (c)
GF1-20	240	237/238	238	96	90/92	92	165	165/167	164/166^g^	247	246/245	245	*Desulfobacterium* (g)
GF1-41	254	254/255	254	92	88/90	88	130	126/127/128	127	463	462	463	*Desulfuromusa* (g)
Paila10-80	270	270/271	269	94	88/90/92	90	163	160/161/162	161	243	242/244	242	*Desulfobulbaceae* (f)
GF1-21	272	270/271	271	96	90/92	92	165	162/165	164	332	332	332	*Desulfobacula (g)*
GF1-39	272	270/271	271	96	90/92	92	165	162/165	162/163/164^g^	nd	468/470/471	469/471^g^	*Desulfobacula (g)*
GF1-34	423	423	423	96	90/92	92	165	165	164	332	332	332	*Desulfobacula (g)*

T-RF  =  terminal restriction fragment, nd  =  not detected.

a Only those T-RFs that were identified with at least three restriction endonucleases are shown.

b Expected T-RFs derived from virtual digestion of partial (approximately 400–500 bp) 16S rRNA gene clone sequences.

c Observed T-RFs (between 50–700 bp) of 16S rRNA genes derived from terminal restriction fragment length polymorphism analysis of sediment samples.

d Observed T-RFs (between 30–700 bp) of 16S rRNA genes derived from terminal restriction fragment length polymorphism analysis of 16S rRNA gene clones.

e Shift of 0–2 bp between observed T-RFs from sediment samples and from 16S rRNA gene clones was allowed since repeats of restriction enzyme digestions of one 16S rRNA gene clone resulted 0–2 bp difference in lengths of observed T-RFs.

f 16S rRNA gene clone sequences used in virtual digestion were assigned to class level and the lowest rank (c  =  class, o  =  order, s  =  suborder, f  =  family, g  =  genus) using taxonomic Classifier (version 2.2, RDP training set 6) of Ribosomal Database Project (RDP) with 80% confidence threshold [39].

g T-RFs of different size derived from one restriction enzyme digestion of one 16S rRNA gene clone.

h T-RFs of different size derived from repeats of restriction enzyme digestions of one 16S rRNA gene clone.

The effects of underlying spatial structure (geographic location and sediment depth) on relationships between bacterial composition and chemical parameters were determined by partial canonical analysis of principal coordinates (partial CAP) (Figure 2B, Figure S2B, S2D and S2F). Although the bacterial ordination was changed in some extent in the partial analysis, the strongest relationships between bacteria (sulphate and sulphur/iron reducers, *Proteobacteria* and *Cyanobacteria*) and chemical parameters remained when the spatial autocorrelation was taken into account. This suggested that correlations between bacteria and chemical parameters were at least partly direct. Partial CAP, based on T-RFs produced by HaeIII, explained 27% of the variation in bacterial communities.

**Figure 3:**
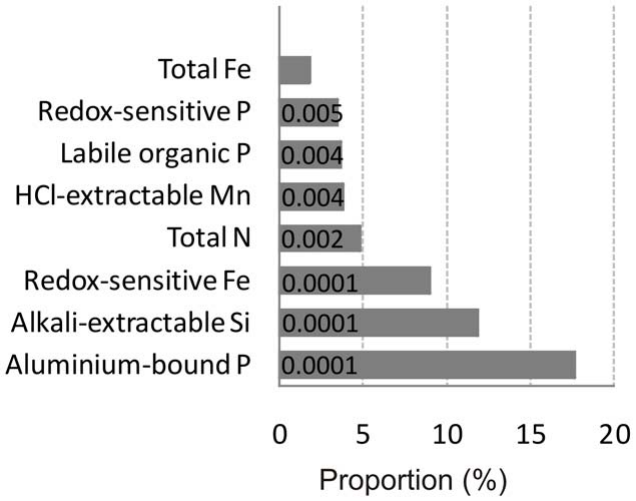
Effects of individual chemical variables on variation in bacterial community composition of Baltic Sea sediments. Proportions were derived from the distance-based multivariate multiple regression analysis of chemical parameters and HaeIII terminal restriction fragments of 16S rRNA genes. *P* values (<0.05) of the forward selection procedure (conditional tests) are shown on the bars.

Multivariate regression analysis specified those chemical parameters (of all chemical parameters used in CAP) which explained most of the variation in bacterial community composition data. Multivariate regression analysis elucidated that among chemical parameters, aluminium-bound phosphorus and alkali-extractable silicon, as well as redox-sensitive iron explained most of the variation in bacterial communities of sediments (Figure 3).

### Proportional effects of spatial, environmental and chemical factors on bacterial communities

**Figure 4:**
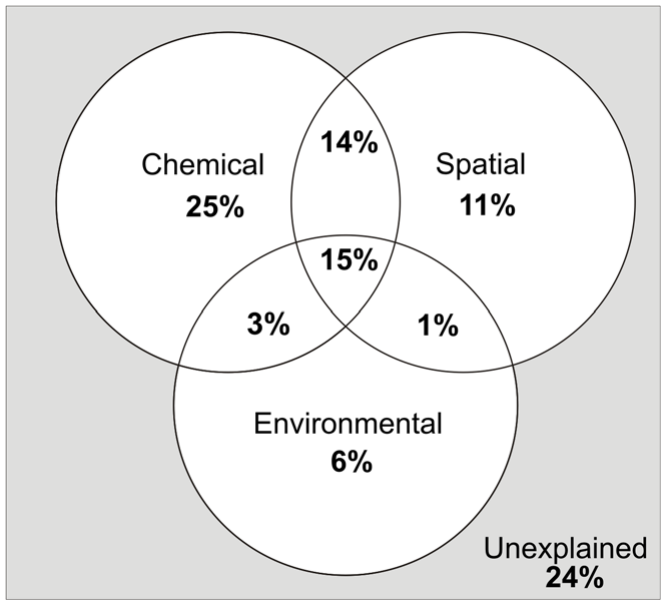
Partitioning of the variation in bacterial community composition between chemical, spatial and environmental variables. Proportions were derived from series of redundancy analysis of terminal restriction fragments (T-RFs) and chemical, spatial and environmental parameters (refer to table S3), which were used in variance partitioning according to Borcard *et al.* [50], Anderson and Gribble [48], and Legendre and Legendre [51]. Spatial parameters were geographic location and sediment depth and environmental parameters included sediment accumulation rate and water depth.

Since bacterial community composition was spatially structured in CAP, the proportional effect of spatial (geographic location and sediment depth) and environmental parameters including sediment accumulation rate and water depth were investigated using variance partitioning. Variance partitioning showed that the chemical and spatial parameters (latitude, longitude and sediment depth) explained 25 and 11% of the variation in bacterial communities (Figure 4), respectively. The environmental parameters, sediment accumulation rate and water depth accounted for 6% of the variation (Figure 4). Sediment accumulation rate and water depth correlated strongly with geographic location (latitude) (Spearman's rho correlations −0,78 and 0,9 respectively) since in the shallow estuary sediment accumulation rate was higher than in the deeper open sea areas. The effects of chemical, spatial and environmental parameters partly overlapped (Figure 4).

### Bacterial taxa in north-eastern Baltic Sea sediments

**Figure 5:**
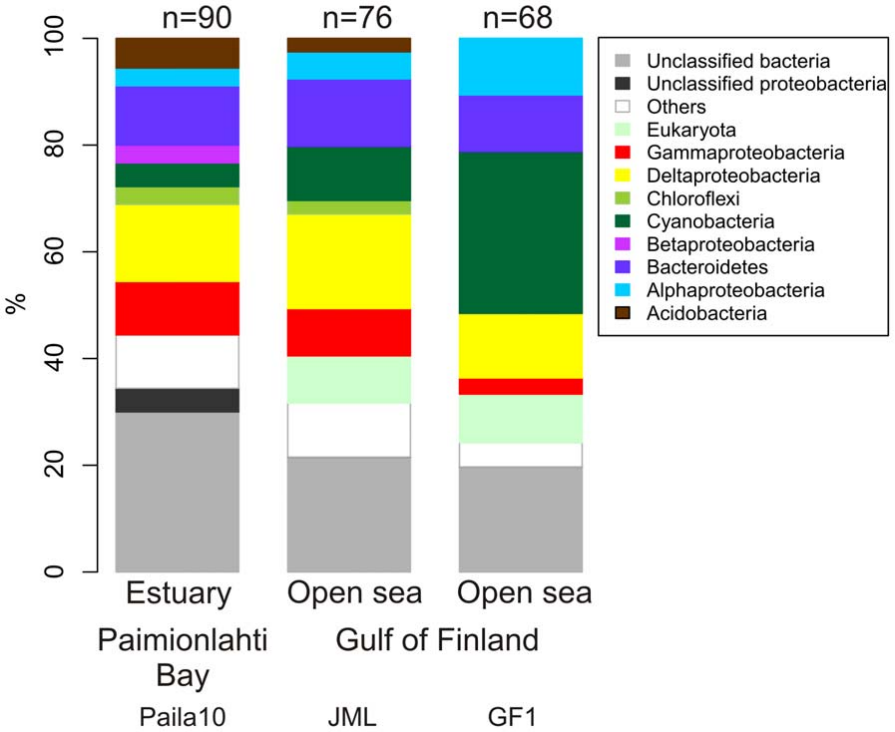
Taxonomic distribution of 16S rRNA gene clones derived from the Baltic Sea sediment clone libraries. Clones were assigned using taxonomic classifier (version 2.2, RDP training set 6) of Ribosomal Database Project (RDP) with confidence threshold of 80%.

To identify the bacterial taxa derived from the Baltic Sea sediment, we constructed three 16S rRNA gene clone libraries (Figure 5) from the surface sediment (0–1 cm) of the estuary (Paimionlahti Bay, Station Paila10) and the open sea (Western Gulf of Finland, stations JML and GF1) (Figure 1A and Figure 1B). Class *Betaproteobacteria* occurred only in the 16S rRNA gene clone library of the estuary (Figure 5). In addition, *Acidobacteria* were most abundant in the estuary. *Alphaproteobacteria* and *Cyanobacteria* occurred in all libraries but were most abundant in the open sea (western Gulf of Finland, station GF1). Especially *Cyanobacteria* were highly abundant on the station GF1.

**Figure 6:**
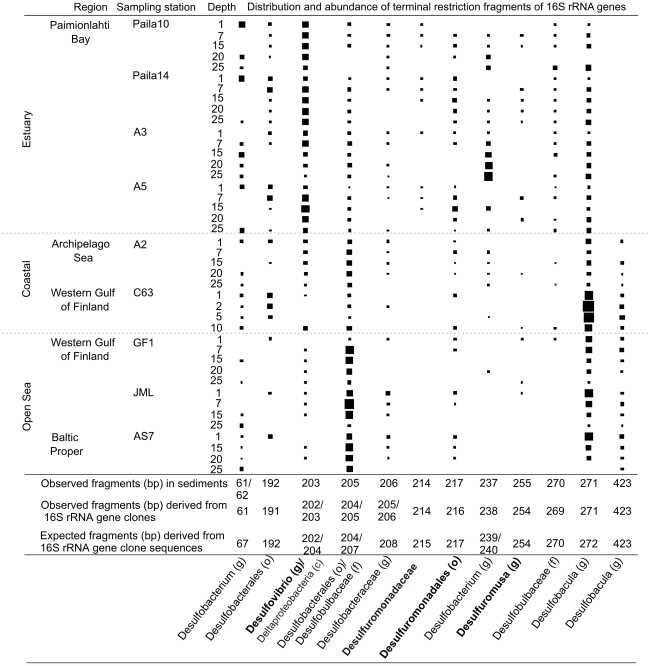
Distribution and relative abundance of potential sulphate- and sulphur/iron-reducing taxa in Baltic Sea sediments. Size of the symbols (squares) corresponds to abundance of a 16S rRNA gene terminal restriction fragment (T-RF, indicated by the same number in bp as in Figure 2) in a sediment sample. The T-RFs were taxonomically assigned, based on *in silico* (virtually) and *in vitro* digested 16S rRNA gene clones (refer to Table 2). Letter c indicates the class, o the order, f the family, and g the genus. Potential iron reducers marked in bold.

*Deltaproteobacteria* sequences were abundant in all three clone libraries (Figure 5). The majority of the *Deltaproteobacteria* clones were assigned to sulphate or sulphur/iron reducing genera (Table S5). Their occurrence varied over the study area (Figure 6, Table 2). Sulphate reducers were particularly common in the coastal and open sea sediments, whereas potential sulphur/iron reducers were more abundant in the iron rich estuary sediments (Paimionlahti Bay). Based on the T-RF data, the family *Desulfuromonadaceae* occurred merely in the estuary sediment. In addition, the genera *Desulfovibrio*, *Desulfobacterium* and *Desulfuromusa* as well as the family *Desulfobulbaceae* were most abundant in the estuary. The genus *Desulfobacula* and bacteria of the order *Desulfobacterales* and the family *Desulfobulbaceae* were abundant in the coastal and open sea sediments (Gulf of Finland, Figure 6). T-FR data thus demonstrated that abundance of various sulphate and sulphur/iron reducing taxa (Figure 6) varied in different sediment habitats of the Baltic Sea.

## Discussion

We found that bacterial communities differed horizontally from the Paimionjoki river estuary to the open sea (the Baltic Proper, Western Gulf of Finland) and vertically from the surface to deeper sediment layers, mainly along the gradient of different phosphorus forms and elements involved in its cycling. The result suggests that phosphorus and its different forms impacted on the bacterial community composition in brackish sediments receiving riverine nutrient loading.

The change in the bacterial community composition was most probably affected by prevailing environmental factors such as oxygen conditions and amount and quality of organic matter along with varying bioavailability of different forms of phosphorus (Table 1). Sequential extraction coarsely separates phosphorus into mobile (labile) and immobile forms but their actual bioavailability depends on the environmental conditions. For instance, bioavailability of iron-bound phosphorus, dominant in sediments of the Paimionjoki river estuary, depends for example on oxygen conditions, pH, and ionic strength, which affect the sorption capacity of phosphorus to the particle or mineral surfaces such as iron oxyhydroxides [52], [53]. Organic phosphorus compounds bound to hydrated oxides of iron via their phosphate groups can be released in poor oxygen conditions thus being more available for microbial degradation [54]. In addition, ability to dissolve both iron-bound and labile organic phosphorus compounds varies among different bacterial species. Thus, it is expectable that variation in bioavailability of different chemical forms of phosphorus can alter bacterial community composition along with other environmental factors. However, a possibility that correlations between bacterial community composition and various phosphorus forms would be only an indication of parallel changes could not be excluded.

Phosphorus forms in a recipient waterbody depend on the amount and quality of loading and on the sedimentation environment. Here, the runoff from the drainage area is rich in particulate phosphorus which can be bioavailable in considerable amounts [24]. It is also well known that humic and particulate material aggregates and settles down depositing also phosphorus bound to their iron oxyhydroxide coatings and iron-phosphorus complexes when riverine freshwater meets saline estuary water [55], [56]. Thus, the amount and quality of the external phosphorus loading probably partly impact the bacterial community composition in recipient sediments via phosphorus chemistry.

To our knowledge, the relationships between bacterial communities and different phosphorus forms have not been investigated to date. In a previous study, the effect of total sediment phosphorus on actively growing bacterial communities was nonsignificant (*P*≥0.005 was regarded nonsignificant) [18]. However, our study showed that different phosphorus forms instead of total phosphorus, e.g. labile organic phosphorus significantly (*P* = 0.004) associated with the bacterial community composition. The total phosphorus does not necessarily reflect feedback interactions between bacterial community composition and phosphorus chemistry since the proportions of mobile (reactive) phosphorus forms in the total phosphorus pool can vary substantially in different sediments [31], [11]–[13]. This is reasonable because bacteria generally utilize the mobile forms of phosphorus more easily than the immobile forms (Table 1).

Correlations between chemical parameters and particular bacteria indicated possible feedback interactions such as microbe-mediated release of phosphorus. Candidates contributing the phosphorus release, sulphate reducers, were abundant in the hypoxic coastal and open sea sediments where the high release of sediment phosphorus took place on certain sites (Table S1). Near-bottom water phosphate concentrations were higher in the open sea than in the estuary area indicating phosphorus release from hypoxic sediments [11]–[13].T-RFs of sulphate reducers such as of genus *Desulfobacula* were associated with elevated concentrations of labile organic phosphorus and total nitrogen (mostly organic nitrogen, [57]), and alkali-extractable silicon which is an indication of biogenic material. The relationship suggests that sulphate reducers contribute to the release of sediment phosphorus via mineralization of labile organic matter with concomitant sulphate reduction. Labile organic matter can be mineralized also in anoxic conditions [58], [54]. However, the relationship can be also a sign of preservation of organic matter in sediments since in low or oscillating redox conditions the degradation rate of organic matter can be retarded and aerobic degradation prevented [58]–[59], [10].

**Figure 7:**
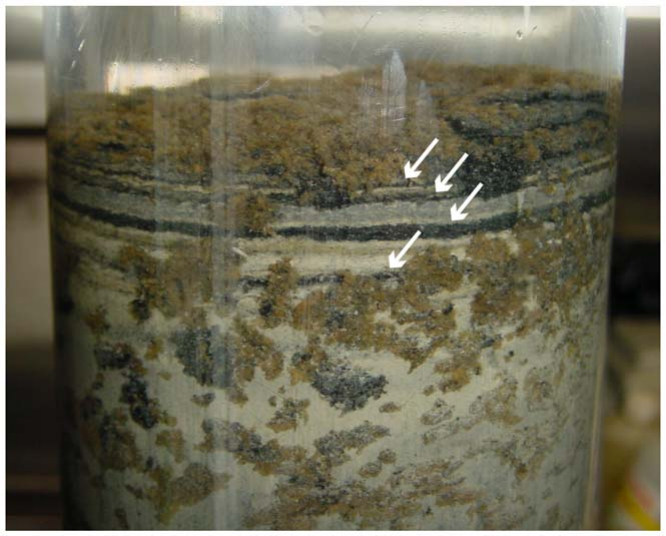
A sediment core from the western Gulf of Finland. The core was sampled from the station JML. Black layers (white arrows) indicate presence of ferrosulphides which are formed via reaction of ferro-iron and sulphite, produced by sulphate reducing bacteria.

The mechanism by which sulphate reducers can participate in phosphorus release is probably indirect. In the open sea sediments (Western Gulf of Finland and Baltic Proper), sulphate reducers may have contributed to high amount of ferrosulphides (Figure 7) in sediments by producing hydrogen sulphide, which precipitates insoluble ferrosulphide (FeS_2_) with iron [60]. As a consequence, iron is not available for binding phosphorus at the sediment surface [61] which enhances phosphorus release from sediment into the water column in anoxic conditions.

The relationships of sulphate reducers with labile organic phosphorus and nitrogen are consistent with the results of earlier studies which showed that sulphate reducers can account for up to 50% of organic matter degradation in coastal marine sediments [62]–[63]. In addition, active sulphate reducers have been detected from coastal sediments of the Baltic Sea [18].

The release of iron-bound phosphate with increasing salinity (and sulphate) concentrations has been described in several estuarine and coastal areas, for example by Hyachinthe and Van Capellen [64] (sediment pore water 0.78±0.15 - 10.92±1.37 psu) and Jordan *et al*. [65] (water column 4–14 and sediment pore water 0–7.5 psu). However, the salinity gradient in bottom water in our study area was weaker (6.2–8.6 psu) than in the previously studied research areas. In low sulphate environments, sulphate concentrations limits sulphate reduction more than in high sulphate environments [66] such as marine sediments where availability of organic substrates limits sulphate reduction rates [62]. Thus we considered that in our study, in the sediments with intermediate salinity, hypoxia and usable organic compounds such as labile organic phosphorus may have raised the abundance of sulphate reducers along with slight salinity changes.

In the estuary (Paimionlahti Bay) sulphate reducers linked to iron reduction such as the genus *Desulfovibrio* [67], [68] and sulphur/ferric iron reducers such as the order *Desulfuromonadales* [69] were abundant. The order *Desulfuromonadales* correlated positively with redox-sensitive, i.e. iron-bound phosphorus and redox-sensitive iron in surface sediments. The genus *Desulfovibrio* was associated with elevated concentrations of total iron and aluminium-bound phosphorus in deeper sediments, 7–15 cm below the seafloor. In addition, the estuary sediments were rich in iron-bound phosphorus and iron which ferric iron reducers can use as the terminal electron acceptor. Reduction of ferric iron (which binds phosphorus) to ferrous iron leads to dissolution of poorly crystallised ferric oxyhydroxides and subsequently release of phosphorus from sediment. Thus, this further supports our conclusion that bioavailability of phosphorus compounds along with other environmental factors possibly affected bacterial communities. Bacteria capable to reduce iron could benefit of high concentration of iron and iron-bound phosphorus in estuary sediments. The association with aluminium-bound phosphorus may be a sign of indirect feedback interaction between unknown factor and sulphur or iron reduction, since aluminium-bound phosphorus is considered immobile in sediments. However, in addition to aluminium-bound phosphorus alkali-extractable fraction may have included some iron-bound phosphorus due to limitations of the sequential extraction method used [11]. This may partly explain the relationship of potential iron reducer (*Desulfovibrio*) and aluminium-bound phosphorus.

Sulphate and sulphur/ferric iron reducing bacteria detected in the deeper sediment layers can also contribute to phosphorus release. However, phosphorus that could be released can be bound again onto hydrated oxides of Al or those of ferric iron in the sediment surface under the oxic bottom water conditions found in the iron-rich estuary (Paimionlahti Bay). Previous results from the Baltic Sea area [70] showed that sulphate reduction was highest below the sediment surface, at a depth of 5.5 cm. Our study and a previous study [70] suggest that sulphate and sulphur/ferric iron reducing bacteria create a potential for phosphorus release from deeper sediment layers which can occur if the bottom water change to anoxic. This could partly explain the extensive variation in phosphorus release (incubation-derived phosphate flux, S1) in the estuary area [11] and also in the open sea area [13].

Cyanobacterial clones were abundant in the clone library from the open sea sediment (Western Gulf of Finland, station GF1, sampled in August/September) probably due to a settled cyanobacterial bloom. Intrestingly, a T-RF of *Synechococcus* was associated with redox-sensitive iron especially in the estuary surface sediments that might indicate a switch from autotrophy to heterotrophy under dark. Heterotrophy of *Synechococcus* has been reported lately [71]. In addition *Synechococcus* possess effective iron acquisition mechanisms [72] which might explain their correlation to phosphorus binding redox-sensitive iron. However, whether the detected *Synechococcus* is capable for heterotrophy and its correlation to redox-sensitive iron is biologically significant remain to be seen.

Also other environmental factors than chemical, such as sediment accumulation rate and water depth, can affect sediment bacterial community composition since the quantity and quality of deposited phosphorus and other nutrients are dependent on sediment accumulation rate and water depth [11], [73]. Sediment accumulation rate and water depth correlated strongly with the geographic location (latitude). Thus, sediment accumulation rate and water depth probably partly explained the geographic variation found. In the estuary (Paimionlahti Bay) sediment accumulation rate is notably higher than in the open sea areas [74]. Variance partition showed that the chemical, spatial and environmental parameters explained 25, 11, and 6% of the variation in the bacterial communities, respectively. We found that sediment accumulation rate and water depth affected only little bacterial community composition. However, our study showed that spatial parameters impacted bacterial community composition but chemical parameters, including different forms of phosphorus, affected even more owing to their varying availability to bacteria.

Our study provided correlative evidence that different chemical forms of phosphorus and elements involved in its cycling, especially iron, affects bacterial community composition due to their varying bioavailability. Especially the occurrence of bacteria which were potentially capable to enhance the availability of phosphorus increased. Such bacteria can contribute to the release of phosphorus, for example, by increasing dissolution of iron-bound phosphorus either directly as iron reducing bacteria or indirectly as sulphate reducing bacteria. However, mechanisms of bacteria-mediated phosphorus cycling remain still to be studied. The mechanisms are essential to take into account when estimating potential to phosphorus release from sediments. Contribution of bacterial activity such as sulphate and iron reduction to phosphorus release in organic rich sediments should be taken into consideration when planning and implementing protective operations to reduce the eutrophication of coastal marine ecosystems.

## Supporting Information

Figure S1 **A preliminary model of canonical analysis of principal coordinates showing relationships between bacterial communities and chemical parameters.** (A) Canonical analysis of principal coordinates (CAP) and (B) partial CAP (spatial autocorrelation was excluded). Samples (n = 42) and HaeIII digested terminal restriction fragments (T-RFs, n = 104) and chemical parameters (red arrows, n = 18) were plotted against canonical axis scores 1 and 2. Black axes correspond to scores of samples/T-RFs and red axes to scores of chemical parameters. T-RFs and their corresponding taxonomic assignments are indicated with purple numbers (in bp) and letters. T-RFs of 16S rRNA gene were identified by digestion of cloned 16S rRNA genes (refer numbers to Table 2 and Table S4). Taxonomic assignments of T-RFs: Ac = *Actinobacteria*, α = *Alphaproteobacteria*, An = *Anaerolineae*, Bac =  *Bacteroidetes*, c = *Cyanobacteria*, δ = *Deltaproteobacteria*, F = *Firmicutes*, γ = *Gammaproteobacteria*, P = *Planctomycetasia*, Pr = *Proteobacteria*, SRB = Sulphate reducing bacteria (*Deltaproteobacteria*), S = Sulphur/iron-reducing bacteria (*Deltaproteobacteria*). Only T-RFs with canonical scores above ±1 for axis 1 and 2 were included. Scores were derived from canonical correlations. The direction of an arrow indicates the increasing concentration of the chemical parameter. The arrow length indicates the strength of the correlation between the corresponding chemical parameter and sediment samples or T-RFs. Chemical parameters: NaClSi  =  loosely bound and pore-water silicon (Si), NaBDiP  =  iron-bound (redox-sensitive inorganic) phosphorus (P), NaBDFe  =  redox-sensitive iron (Fe), NaBDAl  =  NaBD extractable aluminium (Al), NRP  =  labile organic P, NaOHiP  =  Aluminium-bound (alkali-extractable) P, NaOHFe  =  alkali-extractable Fe, NaOHSi  =  alkali-extractable Si, NaOHAl  =  alkali-extractable Al, HCliP  =  apatite P (HCl-extractable), HClMn  =  HCl-extractable manganese (Mn). TN  =  Total nitrogen (N), TC  =  Total carbon (C), TS  =  Total sulphur (S), Ca = calsium. *Position of T-RFs 271 bp was changed for technical reasons. The real canonical scores of the T-RF 271 bp in CAP (A) for axis 1 and 2 were -3.2 and 8.5, and in partial CAP (B) 2.1 and -6.4.
(TIF)

Figure S2 **Relationships between bacterial community composition and chemical parameters of Baltic Sea sediments.** (A, C and E) Canonical analysis of principal coordinates (CAP) and (B, D and F) partial canonical analysis of principal coordinates (partial CAP, spatial autocorrelation was excluded), where samples and chemical parameters (red arrows) were plotted against canonical axis scores 1 and 2. Bacterial communities were determined using terminal restriction fragments (T-RFs) of 16S rRNA genes produced by (A,B) HhaI, (C, D) MspI and (E, F) RsaI restriction enzyme. Black and red axes correspond to axis scores of samples and of chemical parameters, respectively. Chemical parameters: NaBDiP  =  iron-bound (redox-sensitive) phosphorus (P), NaBDFe  =  redox-sensitive iron (Fe), NaOHiP  =  aluminium-bound (alkali-extractable) P, NaOHSi  =  alkali-extractable silicon (Si), NRP  =  labile organic P, and HClMn  =  HCl-extractable manganese (Mn). TN  =  Total nitrogen (N).*Position of the sediment sample was changed for technical reasons. The real canonical scores were 0.8 for axis 1 and -8.2 for axis 2.
(TIF)

Table S1 Properties of the sampled sediments and upperlying bottom water.
(DOC)

Table S2 A sequential phosphorus fractionation scheme.
(DOC)

Table S3 The constrained and/or partial RDA runs used in variance partitioning.
(DOC)

Table S4 Identification of 16S rRNA gene terminal restriction fragments from Baltic Sea sediments.
(DOC)

Table S5 Closest hits of RDP sequences to the 16S rRNA gene clone sequences from Baltic Sea sediments.
(DOC)

Dataset S1 Abundance of terminal restriction fragments produced by HaeIII, HhaI, MspI, and RsaI in the studied sediment samples.
(XLS)

Dataset S2 Concentrations of the chemical parameters used in statistical analyses.
(DOC)
