# Supplementary figures and images for: Phosphorus Chemistry and Bacterial Community Composition Interact in Brackish Sediments Receiving Agricultural Discharges
Source: PLoS One. 2011 Jun 29;6(6):e21555. doi: 10.1371/journal.pone.0021555 (PMC3126828; doi:10.1371/journal.pone.0021555)

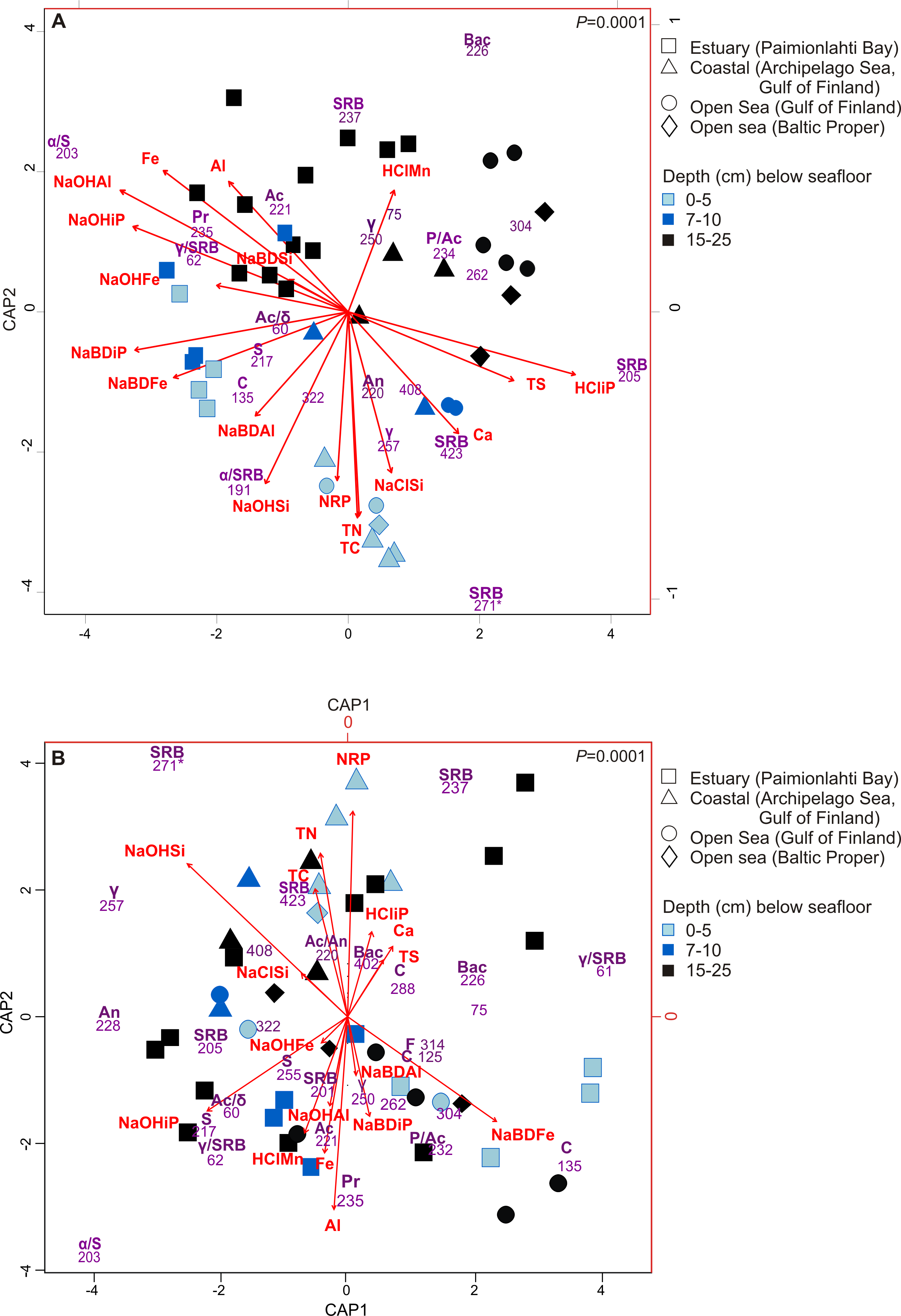

Supplement: Figure S1 — A preliminary model of canonical analysis of principal coordinates showing relationships between bacterial communities and chemical parameters. (A) Canonical analysis of principal coordinates (CAP) and (B) partial CAP (spatial autocorrelation was excluded). Samples (n = 42) and HaeIII digested terminal restriction fragments (T-RFs, n = 104) and chemical parameters (red arrows, n = 18) were plotted against canonical axis scores 1 and 2. Black axes correspond to scores of samples/T-RFs and red axes to scores of chemical parameters. T-RFs and their corresponding taxonomic assignments are indicated with purple numbers (in bp) and letters. T-RFs of 16S rRNA gene were identified by digestion of cloned 16S rRNA genes (refer numbers to Table 2 and Table S4). Taxonomic assignments of T-RFs: Ac = Actinobacteria, α = Alphaproteobacteria, An = Anaerolineae, Bac = Bacteroidetes, c = Cyanobacteria, δ = Deltaproteobacteria, F = Firmicutes, γ = Gammaproteobacteria, P = Planctomycetasia, Pr = Proteobacteria, SRB = Sulphate reducing bacteria (Deltaproteobacteria), S = Sulphur/iron-reducing bacteria (Deltaproteobacteria). Only T-RFs with canonical scores above ±1 for axis 1 and 2 were included. Scores were derived from canonical correlations. The direction of an arrow indicates the increasing concentration of the chemical parameter. The arrow length indicates the strength of the correlation between the corresponding chemical parameter and sediment samples or T-RFs. Chemical parameters: NaClSi = loosely bound and pore-water silicon (Si), NaBDiP = iron-bound (redox-sensitive inorganic) phosphorus (P), NaBDFe = redox-sensitive iron (Fe), NaBDAl = NaBD extractable aluminium (Al), NRP = labile organic P, NaOHiP = Aluminium-bound (alkali-extractable) P, NaOHFe = alkali-extractable Fe, NaOHSi = alkali-extractable Si, NaOHAl = alkali-extractable Al, HCliP = apatite P (HCl-extractable), HClMn = HCl-extractable manganese (Mn). TN = Total nitrogen (N), TC = Total carbon (C), TS = Total sulphur ( [file pone.0021555.s001.tif]

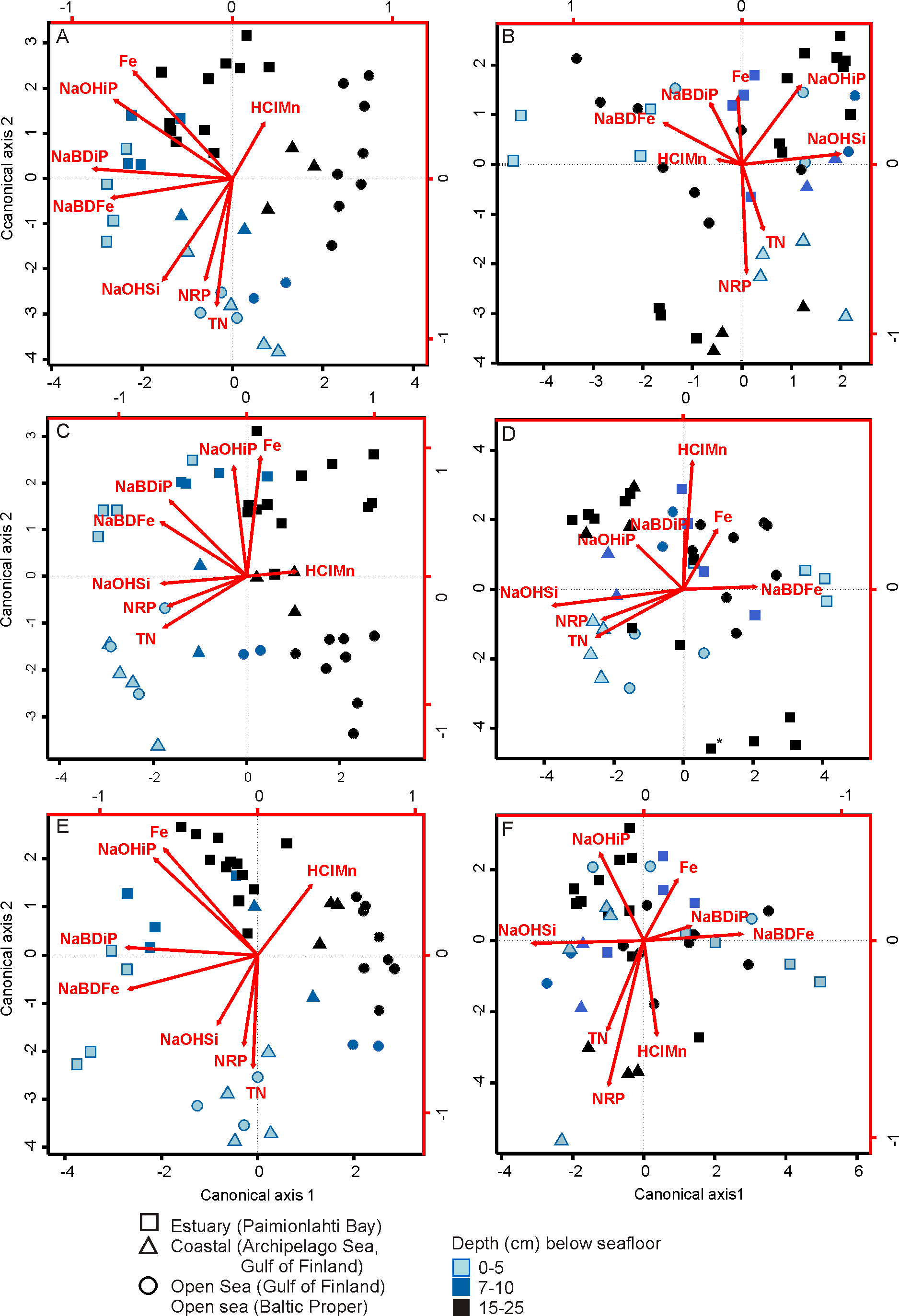

Supplement: Figure S2 — Relationships between bacterial community composition and chemical parameters of Baltic Sea sediments. (A, C and E) Canonical analysis of principal coordinates (CAP) and (B, D and F) partial canonical analysis of principal coordinates (partial CAP, spatial autocorrelation was excluded), where samples and chemical parameters (red arrows) were plotted against canonical axis scores 1 and 2. Bacterial communities were determined using terminal restriction fragments (T-RFs) of 16S rRNA genes produced by (A,B) HhaI, (C, D) MspI and (E, F) RsaI restriction enzyme. Black and red axes correspond to axis scores of samples and of chemical parameters, respectively. Chemical parameters: NaBDiP = iron-bound (redox-sensitive) phosphorus (P), NaBDFe = redox-sensitive iron (Fe), NaOHiP = aluminium-bound (alkali-extractable) P, NaOHSi = alkali-extractable silicon (Si), NRP = labile organic P, and HClMn = HCl-extractable manganese (Mn). TN = Total nitrogen (N).*Position of the sediment sample was changed for technical reasons. The real canonical scores were 0.8 for axis 1 and -8.2 for axis 2. (TIF) [file pone.0021555.s002.tif]
