# Supplementary material for: Phosphorus Chemistry and Bacterial Community Composition Interact in Brackish Sediments Receiving Agricultural Discharges
Source: PLoS One. 2011 Jun 29;6(6):e21555. doi: 10.1371/journal.pone.0021555 (PMC3126828; doi:10.1371/journal.pone.0021555)
Supplement: Table S1 — Properties of the sampled sediments and upperlying bottom water. (DOC) [file pone.0021555.s003.doc]

**Table S1** Properties of the sampled sediments and upperlying bottom water. Geographical coordinates, water depth, sediment accumulation rate (SAR), oxygen, salinity, and incubation-derived phosphate flux in the near-bottom water, as well as redox potential of Baltic Sea sediment. Data from Lukkari et al. [1-3].

| Area | Sampling stations | Coordinates^a^ | | Water depth (m) | SAR^b^  (g m^-2^ y^-1^) | O_2_  (ml l^–1^) | redox  potential  (mV) ^e^ | | PO_4_ -P flux  (µmol m^-2^ d^-2^) | Salinity  (PSU) |
| --- | --- | --- | --- | --- | --- | --- | --- | --- | --- | --- |
|  |  | latitude | longitude |  |  |  | 1 cm | 7cm |  |  |
| Estuary | Paila10 | 60.2215 | 22.3448 | 12 | 4580 | 6.2 | 340.8 | -2.9 | na | na |
|  | Paila14 | 60.1950 | 22.3130 | 29 | 5540 | 2.9 | 290.5 | 25.3 | 278 | 6.2 |
|  | AS5 | 60.1847 | 22.3003 | 19 | 4650 | 1.7 | 291.8 | -13.7 | -4.2 | 6.3 |
|  | AS3 | 60.1327 | 22.2598 | 33 | 840 | 6.1 | 341.3 | 89.9 | 24.4 | 6.3 |
| Coast | AS2 | 60.0488 | 22.1588 | 47 | 900 | 4.9 | 432.0 | 58.1 | 46 | 6.6 |
|  | C63 | 59.4372 | 24.1270 | 45 | 690^c^ | 8.6 | 391.0 | 286.9 | na | 6.0 |
| Open | AS7 | 59.2800 | 21.5650 | 71 | 840 | 1.6 | 10.21 | -111.0 | 198 | 8.6 |
| sea | JML | 59.3491 | 23.3760 | 79 | 354^d^ | 1.8 | 236.8 | -97.8 | -209 | 8.2 |
|  | GF1 | 59.4231 | 24.4092 | 83 | 800^d^ | nd | 54.8 | -192.7 | 1370 | 8.5 |

nd = Not detectable.

na = Not available

^a^ WGS84 coordinate system

^b^ Sediment accumulation rates originated from Mattila et al. [4].

^c^ Average sediment accumulation rate of the Gulf of Finland were used. Sediment accumulation rate of the sampling site was not available due to strong erosion at this site.

^d^Average sediment accumulation rate of the sampling site from 1995 to 2003.

^e^ Considered only suggestive due to common problems involved in measuring redox potential with electrodes [5].

**References**

1. Lukkari K, Leivuori M, Hartikainen H (2008) Vertical distribution and chemical character of sediment phosphorus in two shallow estuaries in the Baltic Sea. Biogeochemistry 90: 171–191.
2. Lukkari K, Leivuori M, Vallius H, Kotilainen A (2009) The chemical character and burial of phosphorus in shallow coastal sediments in the northeastern Baltic Sea. Biogeochemistry 94: 141–162.
3. Lukkari K, Leivuori M, Kotilainen A (2009) Trends in chemical character and burial of sediment phosphorus from open sea to organic rich inner bay in the Baltic Sea. Biogeochemistry 96: 25-48.
4. Mattila J, Kankaanpää H, Ilus E (2006) Estimation of recent sediment accumulation rates in the Baltic Sea using artificial radionuclides ^137^Cs and ^239,240^Pu as time markers. Boreal Env Res 11: 95–107.
5. Drever JI (1997) The Geochemistry of Natural Waters: Surface and Groundwater Environments. New Jersey: Prentice-Hall, Inc.
