# Supplementary material for: Phosphorus Chemistry and Bacterial Community Composition Interact in Brackish Sediments Receiving Agricultural Discharges
Source: PLoS One. 2011 Jun 29;6(6):e21555. doi: 10.1371/journal.pone.0021555 (PMC3126828; doi:10.1371/journal.pone.0021555)
Supplement: Table S2 — A sequential phosphorus fractionation scheme. (DOC) [file pone.0021555.s004.doc]

**Table S2** A sequential phosphorus fractionation scheme. Method is slightly modified from Jensen and Thamdrup [1], by Lukkari et al. [2,3].

| Step | Extractant | Separated P fraction |
| --- | --- | --- |
| I | 0.46 M sodium cloride (NaCl), 1 h | Pore-water P, loosely sorbed P (NaCl-iP) |
| II | 0.11 M sodium dithionite in sodium bicarbonate, pH 7 (NaBD), 1h | P bound to oxides of reducible metals  (Fe and Mn) (NaBD-iP) |
| III | 0.1 M sodium hydroxide (NaOH), 18 h | P from Al oxides, nonreducible Fe compounds (NaOH-iP) and labile organic P (NRP) |
| IV | 0.5 M hydrochloric acid (HCl), 1 h | Apatite and other inorganic P (HCl-iP) |
| V | 1 M hydrochloric acid (HCl), 16 h | Residual, mainly refractory organic P (Res-P) |

**References**

1. Jensen HS, Thamdrup B (1993) Iron-bound phosphorus in marine sediments as measured by bicarbonate-dithionite extraction. Hydrobiologia 253: 47−59.
2. Lukkari K, Hartikainen H, Leivuori M (2007) Fractionation of sediment phosphorus revisited. I: Fractionation steps and their biogeochemical basis. Limnol Oceanogr: Methods 5: 433−444.
3. Lukkari K, Leivuori M, Hartikainen H (2007) Fractionation of sediment phosphorus revisited: II. Changes in phosphorus fractions during sampling and storing in the presence or absence of oxygen. Limnol Oceanogr: Methods 5: 445−456.
