# Supplementary material for: Phosphorus Chemistry and Bacterial Community Composition Interact in Brackish Sediments Receiving Agricultural Discharges
Source: PLoS One. 2011 Jun 29;6(6):e21555. doi: 10.1371/journal.pone.0021555 (PMC3126828; doi:10.1371/journal.pone.0021555)
Supplement: Table S3 — The constrained and/or partial RDA runs used in variance partitioning. (DOC) [file pone.0021555.s005.doc]

**Table S3** The constrained and/or partial RDA runs used in variance partitioning. T-RF-by-samples matrix was constrained by chemical, environmental, and spatial matrix with or without covariables [1] to determine proportional effects of different parameters on the variation in bacterial communities.

| **Constraining parameters** | **Covariabes^1^**  ^1^ |
| --- | --- |
| Chemical | − |
| Spatial | − |
| Environmental | − |
| Chemical | Spatial |
| Chemical | Environmental |
| Chemical | Spatial & environmental Chemical |
| Spatial | Chemical |
| Spatial | Environmental |
| Spatial | Environmental & chemical |
| Environmental | Chemical |
| Environmental | Spatial |
| Environmental | Chemical & spatial |

^1^ parameters which were partitioned out

**References**

1. Anderson MJ, Gribble NA (1998) Partitioning the variation among spatial, temporal and environmental components in a multivariate data set. Aust J Ecol 23: 158-167.
