# Supplementary material for: Phosphorus Chemistry and Bacterial Community Composition Interact in Brackish Sediments Receiving Agricultural Discharges
Source: PLoS One. 2011 Jun 29;6(6):e21555. doi: 10.1371/journal.pone.0021555 (PMC3126828; doi:10.1371/journal.pone.0021555)
Supplement: Table S4 — Identification of 16S rRNA gene terminal restriction fragments from Baltic Sea sediments. (DOC) [file pone.0021555.s006.doc]

**Table S4** Identification of 16S rRNA gene terminal restriction fragments from Baltic Sea sediment.

| T-RF size (bp) ^a^ with | | | | | | | | | | | | | Identification | | |
| --- | --- | --- | --- | --- | --- | --- | --- | --- | --- | --- | --- | --- | --- | --- | --- |
|  | HaeIII |  |  | HhaI |  |  | MspI |  |  | RsaI |  |  | Class or phylum(p)^f^ | lowest rank^g^ | |
| clone | expected^b^ | observed^c^ | observed^d,e^ | expected^b^ | observed^c^ | observed^d,e^ | expected^b^ | observed^c^ | observed^d,e^ | expected^b^ | observed^c^ | observed^d,e^ |  |  |  |
| JML-65 | 38 | nd | 29 | 340 | 340/342 | 340 | 438 | 437 | 437 | 421 | 418/420/422 | 420 | *Alphaproteobacteria* | *Rhodobacteraceae (f*) | |
| GF1-45 | 38 | nd | 29 | 60 | 54/55 | 54 | 436 | 437 | 436 | 510 | 511/512 | 511 | *Alphaproteobacteria* | *Loktanella* (g) | |
| GF1-69 | 38 | nd | 28 | nd | 512/513 | 512 | 129 | 126/127/128 | 126 | 421 | 418/420 | 418 | *Alphaproteobacteria* | *Rhodobacteraceae* (f) | |
| JML-71 | 38 | nd | 29 | 93 | 88/90 | 89 | 88 | 82/84/86 | 79/83/85^h^ | 309 | 307/309 | 308 | *Bacteroidetes* | *Bacteroidetes* (c) | |
| JML-43 | 38 | nd | 29 | 92 | 88/90 | 88 | 504 | 503/505/506 | 504 | 487 | 487/488/490 | 488 | *Deltaproteobacteria* | *Bacteriovorax* (g) | |
| JML-12 | 38 | nd | 31 | nd | nd | nd | 143 | 140 | 142 | 172 | 170 | 170 | *Flavobacteria* | *Algoriphagus* (g) | |
| JML-58 | 38 | nd | 29 | 91 | 88/90 | 87 | 86 | 82/84 | 82 | 307 | 305/307 | 306 | *Flavobacteria* | *Flavobacterium* (g) | |
| JML-63 | 38 | nd | 29 | 93 | 88/90 | 89 | 88 | 82/84/86 | 81/83/85^h^ | 309 | 309/311 | 309 | *Bacteroidetes* | *Bacteroidetes* (p) | |
| JML-74 | 38 | nd | 31 | 99 | 96 | 96 | 545 | 545/546 | 546 | 315 | 311/314/315 | 314 | *Flavobacteria* | *Winogradskyella* (g) | |
| Paila10-66 | 38 | nd | 27 | 97 | 92 | 93 | nd | 542/545 | 543 | 313 | 309/311 | 311 | *Flavobacteria* | *Flavobacteriaceae*(f) | |
| Paila10-19 | 38 | nd | 29 | 60 | 52/54/55 | 53 | nd | 485/487 | 485 | nd | 641/642 | 640 | *Gammaproteobacteria* | *Haliea* (g) | |
| Paila10-23 | 38 | nd | 28 | 210 | 207/209 | 207 | 144 | 140 | 143 | nd | 649 | 647 | *Gammaproteobacteria* | *Gammaproteobacteria*(c) | |
| JML-93 | 38 | nd | 31 | 60 | 52/54/55 | 53 | 71 | 62 | 64 | nd | 641/642 | 640 | *Gammaproteobacteria* | Haliea (g) | |
| JML-3 | 38 | nd | 29 | 355 | 353/356 | 355 | 76 | 71 | 72 | 99 | 94 | 95 | *Sphingobacteria* | *Sphingobacteriales* (o) | |
| JML-25 | 66 | 60/61/62 | 61 | nd | 581/583 | 581 | 148 | 146/148 | 146 | 79 | 72 | 73 | *Acidobacteria* | Gp23 (g) | |
| GF1-43 | 66 | 62 | 60 | 366 | 364/366/368 | 366 | 276 | 275 | 276 | 77 | 72 | 72 | *Actinobacteria* | *Corynebacterineae* (f) | |
| Paila10-51 | 66 | 60/61/62 | 60 | 93 | 88/90 | 88 | 142 | 140 | 140 | 244 | 242 | 241 | *Deltaproteobacteria* | *Deltaproteobacteria* (c) | |
| JML-54 | 66 | 62/61 | 61/62^h^ | 76 | 72/75 | 70/72/75^h^ | 135 | 226 | 227 | nd | 470/471 | 471 | *Gammaproteobacteria* | *Gammaproteobacteria*(c) | |
| JML-48 | 67 | 62/61 | 61 | 96 | 90/92 | 91 | 165 | 161/162 | 161 | 494 | 495 | 494 | *Deltaproteobacteria* | *Desulfobacterium* (g) | |
| Paila10-45 | 68 | 61/62 | 61 | 78 | 72 | 72 | 138 | 136/137/140 | 138 | nd | 470/471/473 | 471 | *Gammaproteobacteria* | *Gammaproteobacteria*(c) | |
| JML-92 | 68 | 62 | 63 | 78 | 72 | 72 | 138 | 136/137/140 | 138 | 471 | 470/471/473 | 471 | *Gammaproteobacteria* | *Gammaproteobacteria*(c) | |
| JML-70 | 71 | 71 | 65 | 83 | 76 | 78 | 200 | 201 | 199 | 441 | 440 | 440 | *Deltaproteobacteria* | *Deltaproteobacteria* (c) | |
| Paila10-13 | 75 | 71 | 70 | 380 | 378 | 377 | 199 | nd | 196 | nd | nd | 482 | *Acidobacteria* | Gp6 (g) | |
| Paila10-36 | 75 | 71 | 69 | 381 | 378 | 378 | 200 | nd | 197 | nd | 484 | 485 | *Acidobacteria* | Gp6 (g) | |
| GF1-9 | 76 | 71 | 71 | 366 | 362/364 | 363 | 131 | 128/129/130 | 130 | 192 | nd | 190 | *Actinobacteria* | *Conexibacteraceae* (f) | |
| JML-77 | 127 | 126/125 | 125 | nd | 667 | 665 | 128 | 127/128/129 | 128 | 420 | 415/418 | 416 | *Cyanobacteria* | GpIIa (g) | |
| GF1-32 | 130 | 135 | 136 | 330 | 334/336 | 336 | 482 | 489/490/491 | 490 | 413 | 418/420 | 418 | *Cyanobacteria* | GpIIa (g) | |
| GF1-54 | 133 | 130/131 | 128 | 352 | 347/349 | 347 | 130 | 128/129/130 | 129 | 157 | nd | 152 | *Gemmatimonadetes* | *Gemmatimonas* (g) | |
| GF1-49 | 136 | 135 | 136 | 336 | 336 | 337 | 488 | 489/491 | 488/490^h^ | 419 | 418/420 | 418 | *Cyanobacteria* | GpIIa (g)^j^ | |

T-RF = terminal restriction fragment, nd= not detected.

^a^ Only those T-RFs that were identified with at least three restriction endonucleases are shown.

^b^ Expected T-RFs derived from virtual digestion of partial (appromiximately 400-500 bp) 16S rRNA gene clone sequences.

^c^ Observed T-RFs (between 50-700 bp) of 16S rRNA genes derived from terminal restriction fragment length polymorphism analysis of sediment samples.

^d^ Observed T-RFs (between 30-700 bp) of 16S rRNA genes derived from terminal restriction fragment length polymorphism analysis of 16S rRNA gene clones.

^e^ Shift of 0−2 bp between observed T-RFs from sediment samples and from 16S rRNA gene clones was allowed since repeats of restriction enzyme digestions of one 16S rRNA gene clone resulted generally 0-2 bp difference in lengths of observed T-RFs.

^f,g^ 16S rRNA gene clone sequences used in virtual digestion were assigned to class and the lowest rank (c = class, o = order, s = suborder, f = family, g = genus) using taxonomic Classifier (version 2.2, RDP training set 6) of Ribosomal Database Project (RDP) with 80% confidence threshold [1].

^h^ T-RFs of different size derived from one restriction enzyme digestion of one 16S rRNA gene clone.

^I^ T-RFs of different size derived from repeats of restriction enzyme digestions of one 16S rRNA gene clone.

^j^ Closest isolate hit at NCBI genbank with 98-100% max identity was the genus *Synechococcus* (Table S5).

**Table S4** (Continued) Identification of 16S rRNA gene terminal restriction fragments from Baltic Sea sediment.

| T-RF size (bp) ^a^ with | | | | | | | | | | | | | | | | Identification | |
| --- | --- | --- | --- | --- | --- | --- | --- | --- | --- | --- | --- | --- | --- | --- | --- | --- | --- |
|  | HaeIII |  |  | | HhaI |  |  | MspI | |  | |  | RsaI |  |  | Class or phylum(p)^f^ | lowest rank^g^ |
| clone | expected^b^ | observed^c^ | observed^d,e^ | | expected^b^ | observed^c^ | observed^d,e^ | expected^b^ | | | observed^c^ | observed^d,e^ | expected^b^ | observed^c^ | observed^d,e^ |  |  |
| JML-32 | 137 | 135 | 135 | | 337 | 336 | 337 | 489 | | | 487/489 | 489 | 420 | 418/420 | 418 | *Cyanobacteria* | GpIIa (g) |
| JML-94 | 137 | 135 | 135 | | 337 | 334/336 | 336 | nd | | | 487/488 | 488 | 420 | 418/420/422 | 420 | *Cyanobacteria* | GpIIa (g) ^j^ |
| GF1-22 | 140 | 141 | 140 | | 93 | 88/90 | 89 | 88 | | | 84/86/88 | 84/86^h^ | 309 | 307/309/311311 | 309 | *Bacteroidetes* | *Bacteroidetes* (p) |
| Paila10-32 | 145 | 141 | 143 | | 95 | 88/90/92 | 90 | 127 | | | 122 | 123 | nd | 490/492 | 491 | *Deltaproteobacteria* | *Desulfomonile* (g) |
| JML-67 | 178 | nd | 176 | | 92 | 88/90 | 88 | 140 | | | 140 | 139 | 83 | 78/91 | 79 | *Deltaproteobacteria* | *Deltaproteobacteria* (c) |
| JML-37 | 183 | nd | 180 | | 96 | 92 | 92 | 134 | | | 129/130 | 131/133^h^ | 57 | 52 | 50 | *Deltaproteobacteria* | *Desulfobacteraceae* (f) |
| Paila10-30 | 188 | 185/187 | 185 | | 225 | 219 | 221 | 124 | | | 120/122 | 12 | nd | 487/488/490 | 488 | *Acidobacteria* | Gp7 (g) |
| GF1-2 | 189 | 189/191 | 191 | | 337 | 334/336 | 336 | 127 | | | 126/127 | 125 | nd | nd | nd | *Alphaproteobacteria* | *Alphaproteobacteria* (c) |
| GF1-11 | 192 | 189/191 | 190 | | 60 | 52/54/55 | 54 | 402 | | | nd | 402/404^h^ | 421 | 420/422 | 421 | *Alphaproteobacteria* | *Amaricoccus* (g) |
| JML-2 | 192 | 191/193 | 191/192^i^ | | 94 | 88/90/92 | 90 | 163 | | | 162 | 163 | 213 | 211/213 | 212 | *Deltaproteobacteria* | *Desulfobacterales* (o) |
| GF1-17 | 195 | 193/195 | 194 | | 99 | 96 | 96 | 440 | | | nd | 441 | 284 | nd | 283 | *Verrucomicrobiae* | *Luteolibacter* (g) |
| Paila10-5 | 195 | 193/195 | 194 | | 99 | 96 | 96 | 433 | | | nd | 434 | 277 | nd | 276 | *Verrucomicrobiae* | *Luteolibacter* (g) |
| Paila10-57 | 195 | 193/195 | 195 | | 99 | 96 | 96 | 432 | | | 430 | 430 | 276 | nd | 274 | *Verrucomicrobiae* | *Luteolibacter* (g) |
| Paila10-6 | 199 | 195/197 | 196 | | 206 | 205 | 203 | 140 | | | 137/140 | 138/140^h^ | nd | nd | nd | *Gammaproteobacteria* | *Gammaproteobacteria*(c) |
| JML-66 | 199 | 195/197 | 197 | | 206 | 205 | 204 | 140 | | | 140 | 140 | nd | nd | nd | *Gammaproteobacteria* | *Gammaproteobacteria*(c) |
| Paila10-53 | 202 | 199/201 | 200 | | 222 | 219 | 219 | *77* | | | nd | 68 | nd | 487/488 | 487 | *Acidobacteria* | Gp22 (g) |
| JML-55 | 202 | 201/203 | 202 | | 92 | 90/92 | 91 | nd | | | 506/508 | 508 | 487 | 490/492 | 491 | *Deltaproteobacteria* | *Desulfovibrio* (g) |
| GF1-40 | 204 | 201/203 | 203 | | 93 | 88/90/92 | 90 | 174 | | | 170/172 | 172/173/174 | nd | nd | nd | *Alphaproteobacteria* | *Alphaproteobacteria* (c) |
| Paila10-52 | 204 | 203/205 | 203 | | 94 | 88/90/92 | 90 | 143 | | | nd | 143 | nd | 490/492 | 492 | *Deltaproteobacteria* | *Deltaproteobacteria* (c) |
| JML-22 | 204 | 205/206 | 205 | | 92 | 88/90/92 | 90 | nd | | | 510/511/512 | 512 | 225 | 226/227 | 226 | *Deltaproteobacteria* | *Desulfobulbaceae* (f) |
| JML-13 | 205 | 203/205 | 204 | | 193 | 190 | 191 | 188 | | | 186 | 188 | 130 | 127/129/130 | 128  191 | *Planctomycetacia* | *Rhodopirellula* (g) |
| JML-27 | 206 | 201/203 | 203 | | 46 | 55/60 | 57/59^h^ | 452 | | | 450/452/456 | 452 | 119 | 114 | 115 | *Alphaproteobacteria* | *Rhodobacteraceae* (f) |
| Paila10-40 | 206 | 205/206 | 205 | | 36 | nd | 25 | 281 | | | 280 | 280 | nd | nd | nd | *Spirochaetes* | *Spirochaetes* (p) |
| JML-64 | 207 | 205/206 | 204 | | 231 | 227 | 227 | 138 | | | 136/137 | 137 | 81 | 77/78 | 77 | *Deltaproteobacteria* | *Desulfobacterales (o)* |
| JML-5 | 208 | 205/206 | 205 | | 96 | 88/90/92 | 90 | 69 | | | 62 | 62/64^h^ | 247 | 242/243 | 243 | *Deltaproteobacteria* | *Desulfobacteraceae* (f) |
| JML-29 | 208 | 205/206 | 206 | | 379 | 378 | 378 | 165 | | | 165 | 164 | 57 | 52 | 51 | *Deltaproteobacteria* | *Desulfobacteraceae* (f) |
| JML-61 | 210 | 206 | 208 | 231 | | 227 | 229 | 90 | 86/88/90 | | | 86/88^h^ | 113 | nd | 110 | *Sphingobacteria* | *Ferruginibacter* (g) |

T-RF = terminal restriction fragment, nd= not detected.

^a^ Only those T-RFs that were identified with at least three restriction endonucleases are shown.

^b^ Expected T-RFs derived from virtual digestion of partial (appromiximately 400-500 bp) 16S rRNA gene clone sequences.

^c^ Observed T-RFs (between 50-700 bp) of 16S rRNA genes derived from terminal restriction fragment length polymorphism analysis of sediment samples.

^d^Observed T-RFs (between 30-700 bp) of 16S rRNA genes derived from terminal restriction fragment length polymorphism analysis of 16S rRNA gene clones.

^e^ Shift of 0−2 bp between observed T-RFs from sediment samples and from 16S rRNA gene clones was allowed since repeats of restriction enzyme digestions of one 16S rRNA gene clone resulted generally 0-2 bp difference in lengths of observed T-RFs.

^f,g^ 16S rRNA gene clone sequences used in virtual digestion were assigned to class and the lowest rank (c = class, o = order, s = suborder, f = family, g = genus) using taxonomic Classifier (version 2.2, RDP training set 6) of Ribosomal Database Project (RDP) with 80% confidence threshold [1].

^h^ T-RFs of different size derived from one restriction enzyme digestion of one 16S rRNA gene clone.

^i^ T-RFs of different size derived from repeats of restriction enzyme digestions of one 16S rRNA gene clone.

^j^ Closest isolate hit at NCBI genbank with 98-100% max identity was the genus *Synechococcus* (Table S5).

**Table S4** (Continued) Identification of 16S rRNA gene terminal restriction fragments from Baltic Sea sediment.

| T-RF size (bp) ^a^ with | | | | | | | | | | | | | | Identification | |  |
| --- | --- | --- | --- | --- | --- | --- | --- | --- | --- | --- | --- | --- | --- | --- | --- | --- |
|  |  | HaeIII |  | | HhaI |  |  | MspI |  |  | RsaI |  |  | Class or phylum(p)^f^ | lowest rank^g^ |  |
| clone | expected^b^ | observed^c^ | observed^d,e^ | | expected^b^ | observed^c^ | observed^d,e^ | expected^b^ | observed^c^ | observed^d,e^ | expected^b^ | observed^c^ | observed^d,e^ |  |  |  |
| Paila10-17 | 215 | 214/216 | | 214 | 92 | 88/90 | 89 | 130 | 126/127/128 | 127 | 57 | 52 | 51 | *Deltaproteobacteria* | *Desulfuromonadaceae* (f) |  |
| Paila10-65 | 217 | 216/217 | 216 | | 94 | 90/92 | 91 | 132 | 128/129/130 | 129 | 57 | 52/54 | 52 | *Deltaproteobacteria* | *Desulfuromonadales* (o) |  |
| Paila10-24 | 219 | 218/220 | 219 | | 395 | 392/394 | 393 | nd | 450/452/453 | 451 | nd | nd | nd | *Anaerolineae* | *Anaerolineaceae* (f) |  |
| GF1-23 | 221 | 220/221 | 220 | | 360 | 362 | 360 | 164 | 165/162 | 163 | 455 | 450 | 451 | *Actinobacteria* | *Actinomycetales* (o) |  |
| Paila10-49 | 221 | 218/220 | 218 | | 209 | 207/211 | 207 | 432 | 430 | 430 | 120 | nd | 117 | *Betaproteobacteria* | *Betaproteobacteria* (o) |  |
| GF1-15 | 225 | 226/223 | 223 | | 481 | nd | 486 | 160 | 157/160/161 | 157/159/161^h^ | 465 | 468/470/471 | 470 | *Clostridia* | *Clostridiales* (o) |  |
| GF1-35 | 226 | 226/228 | 226 | | 290 | nd | 289 | 86 | 84/86 | 84 | 109 | nd | 105 | *Bacteroidetes* | *Sphingobacteria* (c) |  |
| JML-44 | 228 | 228/229 | 228 | | nd | 549/551 | 551 | 527 | 528 | 530 | 73 | 68 | 68 | *Anaerolineae* | *Anaerolineaceae* (f) |  |
| Paila10-56 | 228 | 228/229 | 228 | | nd | 549/551 | 550 | nd | 528 | 528/529/530^i^ | 73 | 68 | 68 | *Anaerolineae* | Anaerolineaceae (f) |  |
| GF1-10 | 232 | 229/232 | 230 | | 373 | 370/371 | 371 | 79 | 75 | 74 | 454 | 448/451 | 451 | *Actinobacteria* | *Ilumatobacter* (g) |  |
| GF1-50 | 233 | 232/234 | 232 | | 60 | 54/55 | 55 | 283 | 280/283 | 282 | 111 | nd | 108 | *Planctomycetacia* | *Planctomyces* (g) |  |
| GF1-12 | 235 | 234/235 | 234 | | 367 | 364/366/368 | 366 | 138 | 136/137 | 135/137^h^ | 457 | 455/457 | 455 | *Actinobacteria* (p) | *Acidimicrobineae* (so) |  |
| Paila10-60 | 236 | 235/237 | 235 | | 66 | 52/54/55 | 54 | 120 | 120 | 116/118^h^ | nd | 508/511 | 510 | *Proteobacteria* | *Proteobacteria* (p) |  |
| JML-7 | 239 | 237/238 | 238 | | 95 | 90/92 | 91 | 164 | 160/161/162 | 161 | 246 | 245/246 | 245 | *Deltaproteobacteria* | *Desulfobacteraceae* (f) |  |
| GF1-6 | 239 | 237/238 | 238 | | 96 | 90/92 | 92 | 164 | 160/161/ | 161 | 246 | 245/246 | 245  245 | *Deltaproteobacteria* | *Desulfobacterium* (g) |  |
| Paila10-20 | 240 | 235/237 | 236 | | 94 | 88/90/92 | 90 | 209 | 206/209 | 208 | nd | 290 | 289 | *Deltaproteobacteria* | *Deltaproteobacteria* (c) |  |
| GF1-20 | 240 | 237/238 | 238 | | 96 | 90/92 | 92 | 165 | 165/167 | 164/166^h^ | 247 | 246/245 | 245 | *Deltaproteobacteria* | *Desulfobacterium* (g) |  |
| Paila10-33 | 252 | 250/252 | 250 | | 206 | 201/205 | 203 | 140 | 137/140 | 139 | nd | nd | nd | *Gammaproteobacteria* | *Gammaproteobacteria*(c) |  |
| GF1-41 | 254 | 254/255 | 254 | | 92 | 88/90 | 88 | 130 | 126/127/128 | 127 | 463 | 462 | 463 | *Deltaproteobacteria* | *Desulfuromusa* (g) |  |
| JML-34 | 258 | 257/258 | 257 | | 372 | 370/371/374 | 372 | 495 | 496/497 | 497 | nd | nd | nd | *Gammaproteobacteria* | *Gammaproteobacteria*(c) |  |
| GF1-13 | 258 | 257/258 | 257 | | nd | nd | 544 | nd | 450/452/453 | 452 | nd | 475/477 | 476 | *Gammaproteobacteria* | *Gammaproteobacteria*(c) |  |
| Paila10-80 | 270 | 270/271 | 269 | | 94 | 88/90/92 | 90 | 163 | 160/161/162 | 161 | 243 | 242/244 | 242 | *Deltaproteobacteria* | *Desulfobulbaceae* (f) |  |
| GF1-21 | 272 | 270/271 | 271 | | 96 | 90/92 | 92 | 165 | 162/165 | 164 | 332 | 332 | 332 | *Deltaproteobacteria* | *Desulfobacula* (g) |  |
| GF1-39 | 272 | 270/271 | 271 | | 96 | 90/92 | 92 | 165 | 162/165 | 162/163/164^h^ | nd | 468/470/471 | 469/471^h^ | *Deltaproteobacteria* | *Desulfobacula* (g) |  |
| GF1-16 | 289 | 288/290/291 | | 289 | 36 | nd | 28 | nd | 492/494/496 | 494 | 420 | 418/420 | 419 | *Cyanobacteria* | *GpIIa* (g) | |
| GF1-24 | 289 | 298 | | 296 | 140 | nd | 146 | 489 | 494/496/497 | 496 | 420 | 427/429/430 | 429 | *Cyanobacteria* | *GpIIa* (g) |  |

T-RF = terminal restriction fragment, nd= not detected.

^a^ Only those T-RFs that were identified with at least three restriction endonucleases are shown.

^b^ Expected T-RFs derived from virtual digestion of partial (appromiximately 400-500 bp) 16S rRNA gene clone sequences.

^c^ Observed T-RFs (between 50-700 bp) of 16S rRNA genes derived from terminal restriction fragment length polymorphism analysis of sediment samples.

^d^Observed T-RFs (between 30-700 bp) of 16S rRNA genes derived from terminal restriction fragment length polymorphism analysis of 16S rRNA gene clones.

^e^ Shift of 0−2 bp between observed T-RFs from sediment samples and from 16S rRNA gene clones was allowed since repeats of restriction enzyme digestions of one 16S rRNA gene clone resulted generally 0-2 bp difference in lengths of observed T-RFs.

^f,g^ 16S rRNA gene clone sequences used in virtual digestion were assigned to class and the lowest rank (c = class, o = order, s = suborder, f = family, g = genus) using taxonomic Classifier (version 2.2, RDP training set 6) of Ribosomal Database Project (RDP) with 80% confidence threshold [1].

^h^ T-RFs of different size derived from one restriction enzyme digestion of one 16S rRNA gene clone.

^i^ T-RFs of different size derived from repeats of restriction enzyme digestions of one 16S rRNA gene clone.

**Table S4** (Continued) Identification of 16S rRNA gene terminal restriction fragments from Baltic Sea sediment.

| T-RF size (bp) ^a^ with | | | | | | | | | | | | | | | Identification | | | |
| --- | --- | --- | --- | --- | --- | --- | --- | --- | --- | --- | --- | --- | --- | --- | --- | --- | --- | --- |
|  |  | HaeIII |  | HhaI | | |  | MspI | |  | RsaI | | |  | Class or phylum(p)^f^ | | lowest rank^g^ | |
| clone | expected^b^ | observed^c^ | observed^d,e^ | expected^b^ | observed^c^ | | observed^d,e^ | expected^b^ | observed^c^ | observed^d,e^ | expected^b^ | observed^c^ | | observed^d,e^ |  | |  | |
| GF1-55 | 290 | 288/290/291 | 289 | 338 | 334/336 | 336 | | 490 | 491/489 | 488/490^h^ | 421 | 420/422 | 421 | | *Cyanobacteria* | *GpIIa* (g) | | |
| JML-35 | 291 | 290/291 | 290 | --- | 671 | 670 | | 492 | 491/492/494 | 492 | 423 | 422 | 422 | | *Cyanobacteria* | *GpI* (g) | | |
| JML-79 | 291 | 290/291/292 | 291 | 227 | 227 | | 226 | 149 | 148/149 | 149 | 422 | 420/422 | | 422 | *Cyanobacteria* | *GpI* (g) | |  |
| GF1-26 | 291 | 290/291 | 290 | --- | 667 | | 668 | 491 | 491/492/494 | 491/493^h^ | 422 | 420/422 | | 421 | *Cyanobacteria* | *GpIII* (g) | |  |
| GF1-59 | 294 | 292 | 294 | 83 | 76 | | 78 | 131 | 128/129/130 | 130 | 201 | 201 | | 201 | *Alphaproteobacteria* | *Alphaproteobacteria* (c) | |  |
| JML-8 | 298 | 298 | 296 | 60 | 52/54/55 | | 53 | 295 | 294/295 | 295 | 447 | 447/448 | | 445 | *Acidobacteria* | *Gp26* (g) | |  |
| GF1-68 | 298 | 298 | 298 | 87 | − | | 83 | 135 | 136 | 134 | 205 | − | | 204 | *Alphaproteobacteria* | *Alphaproteobacteria* (c) | |  |
| Paila10-38 | 318 | 315/317 | 316 | 66 | 54/55 | | 55 | --- | 485/487/489 | 487 | --- | − | | − | *Proteobacteria* | *Proteobacteria* (p) | |  |
| GF1-74 | 318 | 270/271/272 | 271 | 366 | 364/366/368 | | 366 | 487 | 489/491 | 491 | --- | 495 | | 494 | *Gammaproteobacteria* | *Methylobacter* (g) | |  |
| JML-28 | 321 | 320/321 | 320 | 209 | 207 | | 207 | 492 | 494/486 | 494 | 462 | 462 | | 461 | *Gammaproteobacteria* | *Gammaproteobacteria*(c) | |  |
| Paila10-18 | 330 | 328/329 | 327 | 36 | − | | 27 | --- | 600 | 601 | --- | 471/473/475 | | 473 | *Nitrospira* | *Nitrospira* (g) | |  |
| JML-16 | 380 | 380 | 380 | --- | − | | − | 496 | 496/497 | 497/499^i^ | 57 | 52 | | 51 | *Eukaryota* | *Bacillariophyta* (g) | |  |
| JML-33 | 404 | 402 | 403 | 91 | 88/90 | | 89 | 537 | 538/539/540 | 540 | 307 | 307/309 | | 307 | *Bacteroidetes* | *Bacteroidetes* (p) | |  |
| Paila10-55 | 406 | 406 | 405 | 93 | 88/90 | | 89 | 88 | 82/84/86 | 84 | 111 | − | | 107 | *Sphingobacteria* | *Saprospiraceae* (f) | |  |
| JML-57 | 407 | 408/406 | 406 | 368 | 366/368 | | 367 | 163 | 160/161/162 | 160 | 229 | 227/229 | | 227 | *Lentisphaerae* | *Lentisphaeria* (c) | |  |
| GF1-34 | 423 | 423 | 423 | 96 | 90/92 | | 92 | 165 | 165 | 164 | 332 | 332 | | 332 | *Deltaproteobacteria* | *Desulfobacula* (g) | |  |
| Paila10-64 | 424 | 421 | 422 | 111 | 109 | | 107 | 222 | − | 220 | 129 | 128/129/130 | | 129 | *Sphingobacteria* | *Saprospiraceae* (f) | |  |
| JML-36 | 549 | 550 | − | 376 | 375/378 | | 376 | 95 | 92 | 93 | 118 | 114 | | 115 | *Sphingobacteria* | *Sphingobacteriales* (o) | |  |

T-RF = terminal restriction fragment, nd= not detected.

^a^ Only those T-RFs that were identified with at least three restriction endonucleases are shown.

^b^ Expected T-RFs derived from virtual digestion of partial (appromiximately 400-500 bp) 16S rRNA gene clone sequences.

^c^ Observed T-RFs (between 50-700 bp) of 16S rRNA genes derived from terminal restriction fragment length polymorphism analysis of sediment samples.

^d^Observed T-RFs (between 30-700 bp) of 16S rRNA genes derived from terminal restriction fragment length polymorphism analysis of 16S rRNA gene clones.

^e^ Shift of 0−2 bp between observed T-RFs from sediment samples and from 16S rRNA gene clones was allowed since repeats of restriction enzyme digestions of one 16S rRNA gene clone resulted generally 0-2 bp difference in lengths of observed T-RFs.

^f,g^ 16S rRNA gene clone sequences used in virtual digestion were assigned to class and the lowest rank (c = class, o = order, s = suborder, f = family, g = genus) using taxonomic Classifier (version 2.2, RDP training set 6) of Ribosomal Database Project (RDP) with 80% confidence threshold [1].

^h^ T-RFs of different size derived from one restriction enzyme digestion of one 16S rRNA gene clone.

^i^ T-RFs of different size derived from repeats of restriction enzyme digestions of one 16S rRNA gene clone.

**References**

1. Wang Q, Garrity GM, Tiedje JM, Cole JR (2007) Naïve Bayesian Classifier for Rapid Assignment of rRNA Sequences into the New Bacterial Taxonomy. Appl Environ Microbiol 73: 5261–5267.
