# Supplementary material for: Phosphorus Chemistry and Bacterial Community Composition Interact in Brackish Sediments Receiving Agricultural Discharges
Source: PLoS One. 2011 Jun 29;6(6):e21555. doi: 10.1371/journal.pone.0021555 (PMC3126828; doi:10.1371/journal.pone.0021555)
Supplement: Table S5 — Closest hits of RDP sequences to the 16S rRNA gene clone sequences from Baltic Sea sediments. (DOC) [file pone.0021555.s007.doc]

**Table S5.** Closest hits of RDP sequences (Release 10.10) to the 16S rRNA gene clone sequences from Baltic Sea sediment. GF1 and JML sequences originated from the western Gulf of Finland and P10 from Paimionlahti Bay. Searches were done with the seqmatch tool, version 3 [1,2].

| Sequnce code | Class | Lowest rank | Accession number | Score | Isolation source |
| --- | --- | --- | --- | --- | --- |
| GF1-1 | Unclassified | Bacteria (d) | AF449262 | 0.624 | *Riftia pachyptila* tube |
| GF1-2 | *Alphaproteobacteria* | *Rhodobacteraceae* (f) | EF664527 | 0.921 | Soil |
| GF1-6 | *Deltaproteobacteria* | *Desulfobacterium* (g) | AM746260 | 0.983 | Surface sediment, Baltic Sea |
| GF1-9 | *Actinobacteria* | *Conexibacter* (g) | EF459919 | 0.926 | Sediment, Baltic Sea |
| GF1-10 | *Actinobacteria* | *Actinomycetales* (o) | EF460009 | 0.884 | Sediment, Baltic Sea |
| GF1-11 | *Alphaproteobacteria* | *Amaricoccus* (g) | EF464794 | 0.848 | Soil, Antarctica |
| GF1-12 | *Actinobacteria* | *Actinobacteria* (c) | EU645020 | 0.901 | Meromictic soda lake |
| GF1-13 | *Gammaproteobacteria* | *Gammaproteobacteria (c)* | EF459860 | 0.990 | Sediment, Baltic Sea |
| GF1-14 | Eukaryota | *Bacillariophyta* (g) | AB426417 | 0.833 | deep-sea sediment, microbial mat |
| GF1-15 | *Clostridia* | *Ruminococcaceae* (f) | FJ223391 | 0.812 | marine sediment, bacterial mat, Antarctica |
| GF1-16 | *Cyanobacteria* | GpIIa (g)^a^ | AY151240 | 0.969 | Subalpine lake |
| GF1-17 | *Verrucomicrobiae* | Verrucomicrobiaceae  genera incertae sedis (g) | EU376209 | 0.959 | Lake water column during cyanobacterial bloom |
| GF1-18 | *Cyanobacteria* | GpIIa(g)^a^ | AY151240 | 0.986 | Subalpine lake |
| GF1-19 | *Actinobacteria* | *Micrococcineae* (so) | EF029021 | 0.981 | Reduced sediment associated with *Zostera marina* |
| GF1-20 | *Deltaproteobacteria* | *Desulfobacterium* (g) | AM746260 | 0.983 | Baltic Sea surface sediment |
| GF1-21 | *Deltaproteobacteria* | *Desulfobacula* (g) | AY177803 | 0.861 | Antarctic sediment |
| GF1-22 | *Bacteroidetes* | *Bacteroidales* (o) | EU925908 | 0.856 | Sediment, northern Bering Sea |
| GF1-23 | *Actinobacteria* | *Actinomycetales* (o) | EF471611 | 0.970 | Surface water, Chesapeake Bay |
| GF1-24 | *Cyanobacteria* | GpIIa (g)^a^ | AB154316 | 0.987 | Freshwater, Japan, Lake Kasumigaura |
| GF1-25 | *Cyanobacteria* | GpIIa (g)^a^ | Z77405 | 0.748 | Water from 420 metres, Mediterranean Sea |
| GF1-26 | *Cyanobacteria* | GpIII (g)* | AF132792 | 1.000 |  |
| GF1-27 | Unclassified | OD1 genera incertae sedis (g) | AB015577 | 0.701 | deep-sea sediment |
| GF1-28 | *Cyanobacteria* | GpIIa (g)^a^ | AB154316 | 1.000 | Freshwater, Japan, Lake Kasumigaura |
| GF1-29 | *Cyanobacteria* | GpIIa (g)^a^ | AY151241 | 1.000 | Subalpine lake |
| GF1-30 | *Deltaproteobacteria* | *Deltaproteobacteria* (c) | AM746276 | 0.931 | Surface sediment, Baltic Sea |
| GF1-31 | *Cyanobacteria* | GpIIa (g)^a^ | AY151241 | 1.000 | Subalpine lake |
| GF1-32 | *Cyanobacteria* | GpIIa (g)^a^ | AB154316 | 1.000 | Freshwater, Japan, Lake Kasumigaura |
| GF1-33 | *Cyanobacteria* | GpIIa (g)^a^ | AJ639896 | 1.000 | Bubano Basin, Italy |
| GF1-34 | *Deltaproteobacteria* | *Desulfobacula* (g)^b^ | AJ237606 | 0.853 | marine mud |
| GF1-35 | unclassified | *Bacteroidetes* (p) | DQ861110 | 0.615 | Microbial mat, Western Australia, Shark Bay |
| GF1-36 | *Verrucomicrobiae* | Subdivision 5 genera incertae sedis (g) | AM086141 | 0.536 | Lake profundal sediment, Israel |
| GF1-37 | *Deltaproteobacteria* | *Deltaproteobacteria* (c) | EF516405 | 0.487 | grassland soil, USA: northern California |
| GF1-38 | *Planctomycetacia* | *Planctomycetaceae* (f) | FJ717265 | 0.599 | Marine sediment, Cullercoats, United Kingdom |
| GF1-39 | *Deltaproteobacteria* | *Desulfobacula* (g) | AY177795 | 0.883 | Antarctic sediment |
| GF1-40 | *Alphaproteobacteria* | *Alphaproteobacteria* (c) | FM176237 | 0.785 | Mountain rivulet, Germany |
| GF1-41 | *Deltaproteobacteria* | *Desulfuromusa* (g) | AY327186 | 0.907 | Sulfide-rich spring |
| GF1-42 | *Cyanobacteria* | GpIIa (g ^a^ | AY151241 | 1.000 | Subalpine lake |
| GF1-43 | *Actinobacteria* | *Mycobacterium* (g) | DQ829169 | 0.833 | Agricultural soil |
| GF1-44 | *Cyanobacteria* | GpIIa (g) | EF088334 | 0.749 | Waste stabilization pond |
| GF1-45 | *Alphaproteobacteria* | *Loktanella* (g)^b^ | FJ195992 | 0.954 | Sediment, Antarctic Ocean |
| GF1-46 | *Cyanobacteria* | GpIIa (g) | AY151241 | 0.857 | Subalpine lake |
| GF1-47 | Unclassified | Bacteria (d) | AY711058 | 0.718 | Sediment 12−13 cm, Sapelo Island, USA |
| GF1-48 | *Betaproteobacteria* | *Alcaligenes* (g) | EF471606 | 0.976 | Surface water, Chesapeake Bay |
| GF1-49 | *Cyanobacteria* | GpIIa (g)^a^ | AB154316 | 0.976 | Freshwater, Japan, Lake Kasumigaura |
| GF1-50 | *Planctomycetacia* | *Planctomyces* (g) | EF221172 | 0.961 | Vegetated soil, Antarctica |
| GF1-51 | *Cyanobacteria* | GpIIa (g)^a^ | AY151241 | 1.000 | Subalpine lake |
| GF1-52 | *Cyanobacteria* | GpIIa (g)^a^ | AB154316 | 1.000 | Freshwater, Japan, Lake Kasumigaura |
| GF1-53 | *Actinobacteria* | *Actinobacteria* (c) | DQ520161 | 0.956 | Hypertrophic freshwater lake, China |

^a^ Closest isolate hit at NCBI genbank with 98-100% max identity was the genus *Synechococcus.*

^b^ Closest hit was an isolate

**Table S5.** (Continued) Closest hits of RDP sequences (Release 10.10) to the 16S rRNA gene clone sequences from Baltic Sea sediment. GF1 and JML sequences originated from the western Gulf of Finland and P10 from Paimionlahti Bay. Searches were done with the seqmatch tool, version 3 [1,2].

| Sequnce code | Class | Lowest rank | Accession number | Score | Isolation source |
| --- | --- | --- | --- | --- | --- |
| GF1-54 | *Gemmatimonadetes* | *Gemmatimonas* (g) | EF393220 | 0.924 | River sediment, PCB-spiked, incubated anaerobically |
| GF1-55 | *Cyanobacteria* | GpIIa (g)^a^ | AF448061 | 0.956 |  |
| GF1-56 | *Cyanobacteria* | GpIIa (g)^a^ | AY151238 | 0.955 | Subalpine lake |
| GF1-57 | *Deltaproteobacteria* | *Deltaproteobacteria* (c) | EF459870 | 0.982 | Sediment, Baltic Sea |
| GF1-58 | *Cyanobacteria* | GpIV (g)^b^ | EF654067 | 0.856 | Culture collection strain |
| GF1-59 | *Alphaproteobacteria* | *Rhizobiales* (o) | DQ828860 | 0.964 | Agricultural soil, Switzerland |
| GF1-60 | *Gemmatimonadetes* | *Gemmatimonas* (g) | EU283563 | 0.889 | Anderson Lake, USA |
| GF1-66 | *Gammaproteobacteria* | *Gammaproteobacteria* (c) | FJ665215 | 0.500 | ground swab in mangrove forest, India |
| GF1-67 | Eukaryota | *Bacillariophyta* (g) | FM242286 | 0.637 | Sediment, France |
| GF1-68 | *Alphaproteobacteria* | *Alphaproteobacteria* (c) | AY425764 | 0.927 | Volcanic deposit, USA, Hawaii |
| GF1-69 | *Alphaproteobacteria* | *Rhodobacter* (g) | EU073791 | 0.959 | Coal |
| GF1-70 | Eukaryota | *Bacillariophyta* (g) | AB426417 | 0.854 | deep-sea sediment, microbial mat |
| GF1-71 | Unclassified | Bacteria (d) | AF449263 | 0.755 | *Riftia pachyptila* tube |
| GF1-73 | *Cyanobacteria* | GpIIa (g)^a^ | DQ670081 | 0.768 | Seawater, offshore Chile |
| GF1-74 | *Gammaproteobacteria* | *Methylobacter* (g) | FJ712568 | 0.765 | Mud volcano, Mediterranean Sea |
| GF1-75 | *Deltaproteobacteria* | *Desulfobacula* (g) | FM246562 | 0.900 | Upwelling regime water, Namibia |
| GF1-76 | *Cyanobacteria* | GpI (g) ^b^ | AF268004 | 0.967 | Baltic Sea |
| GF1-78 | *Cyanobacteria* | GpI (g) ^b^ | AY439283 | 0.987 | Baltic Sea |
| GF1-79 | *Cyanobacteria* | GpI (g) ^b^ | AF268004 | 0.965 | Baltic Sea |
| GF1-80 | *Cyanobacteria* | GpI (g) ^b^ | AJ224447 | 0.969 | Baltic Sea |
| JML-1 | Unclassified | *Bacteria* (d) | AY711312 | 0.743 | Sediment, Sapelo Island |
| JML-2 | *Deltaproteobacteria* | *Desulfobulbaceae* (f) | DQ112391 | 0.888 | Intertidal mudflat sediment |
| JML-3 | *Sphingobacteria* | *Sphingobacteriales* (o) | FJ694453 | 0.902 | Freshwater |
| JML-4 | Eukaryota | *Chloroplast* (f) | AY948053 | 0.802 | Parker River water |
| JML-5 | *Deltaproteobacteria* | *Desulfobacteraceae* (f) | EU362228 | 0.858 | Tidal flat sediment |
| JML-6 | *Cyanobacteria* | *Bacillariophyta* (g) | AB426417 | 0.886 | Deep-sea sediment, microbial mat |
| JML-7 | *Deltaproteobacteria* | *Deltaproteobacteria ( c)* | AM746276 | 0.977 | Surface sediment, Baltic Sea |
| JML-8 | *Acidobacteria* | *Gp26* (g) | EU491340 | 0.880 | Seafloor lavas, Hawaii |
| JML-12 | *Flavobacteria* | *Algoriphagus* (g) | EF471633 | 0.941 | Surface water, Chesapeake Bay |
| JML-13 | *Planctomycetacia* | *Planctomycetaceae* (f) | AM409874 | 0.907 | Lake profundal sediment |
| JML-16 | Eukaryota | *Bacillariophyta* (g) | FJ516952 | 0.931 | Wetland sediment |
| JML-18 | *Deltaproteobacteria* | *Desulfobacula* (g) | AY177803 | 0.851 | Antarctic sediment |
| JML-20 | Eukaryota | *Bacillariophyta* (g) | FJ744935 | 0.673 | Marine surface water, Sapelo Island |
| JML-22 | *Deltaproteobacteria* | *Desulfobulbaceae* (f) | AB234257 | 0.762 | Sediment/ soil |
| JML-23 | *Deltaproteobacteria* | *Desulfuromonales (o)* | EF668030 | 0.683 | Uranium bioremediation site |
| JML-24 | Eukaryota | *Bacillariophyta* (g) | Z77511 | 0.657 | Mediterranean Sea water |
| JML-25 | *Acidobacteria* | *Gp23* (g) | AB240699 | 0.852 | Cold seep sediment |
| JML-26 | *Cyanobacteria* | *GpIIa* (g) | AB154316 | 1.000 | Freshwater, Japan, Lake Kasumigaura |
| JML-27 | *Alphaproteobacteria* | *Rhodobacteraceae* (f) | EF460023 | 0.922 | Sediment, Baltic Sea |
| JML-28 | *Gammaproteobacteria* | *Gammaproteobacteria* (c) | EF459911 | 0.873 | Sediment, Baltic Sea |
| JML-29 | *Deltaproteobacteria* | *Desulfobacteraceae* (f) | EF459882 | 0.700 | Sediment, Baltic Sea |
| JML-30 | *Spirochaetes* | *Spirochaeta* (g) | AB177064 | 0.830 | Sediment, Pacific Ocean Margin |
| JML-31 | *Cyanobacteria* | *Bacillariophyta* (g) | EF395719 | 0.920 | Surface water, Chesapeake Bay |
| JML-32 | *Cyanobacteria* | *GpIIa* (g) | AM710358 | 1.000 | Freshwater, Machovo jezero |
| JML-33 | *Bacteroidetes* | *Alkaliflexus* (g) | EU735602 | 0.788 | Oil-contaminated soil, China |
| JML-34 | *Gammaproteobacteria* | *Gammaproteobacteria* (c) | EF459860 | 0.945 | Sediment, Baltic Sea |
| JML-35 | *Cyanobacteria* | *GpI* (g) | EF568908 | 1.000 | Baltic Sea water, plankton |
| JML-36 | *Sphingobacteria* | *Saprospiraceae* (f) | EU542170 | 0.726 | Roots of *Zostera marina*, Chesapeake Bay |
| JML-37 | *Deltaproteobacteria* | *Desulfosarcina* (g) | DQ351771 | 0.772 | Marine sediments, North Sea |
| JML-38 | *Nitrospira* | *Magnetobacterium* (g) | FJ516996 | 0.949 | Water, wetland |

^a^ Closest isolate hit at NCBI genbank with 98-100% max identity was the genus *Synechococcus.*

^b^ Closest hit was an isolate

**Table S5.** (Continued) Closest hits of RDP sequences (Release 10.10) to the 16S rRNA gene clone sequences from Baltic Sea sediment. GF1 and JML sequences originated from the western Gulf of Finland and P10 from Paimionlahti Bay. Searches were done with the seqmatch tool, version 3 [1,2].

| Sequnce code | Class | Lowest rank | Accession number | Score | Isolation source |
| --- | --- | --- | --- | --- | --- |
| JML-39 | Unclassified | *Bacteria* (d) | EU245178 | 0.738 | Hypersaline microbial mat |
| JML-42 | Unclassified | *Bacteria* (d) | AB186808 | 0.910 | Polychlorinated dioxin-dechlorinating microcosm |
| JML-43 | *Deltaproteobacteria* | *Bacteriovorax* (g) | FM214334 | 0.782 | Surface marine sediment, Antarctica |
| JML-44 | *Anaerolineae* | Anaerolinea (g) | FM206155 | 0.724 | Chlorinated ethene-contaminated groundwater |
| JML-45 | Unclassified | *Bacteria* (d) | FJ416124 | 0.882 | Sediment, northern Bering Sea |
| JML-46 | *Gammaproteobacteria* | *Gammaproteobacteria* (c) | EF459874 | 0.980 | Sediment, Baltic Sea |
| JML-47 | Unclassified | *Bacteria* (d) | FJ712494 | 0.814 | Mud volcano, Mediterranean Sea |
| JML-48 | *Deltaproteobacteria* | Desulfobacterium (g)^b^ | U85471 | 0.778 | Salt marsh sediment |
| JML-49 | *Actinobacteria* | Rubrobacterineae (so) | EF520360 | 0.613 | Acidic deposition-impacted lake |
| JML-50 | *Alphaproteobacteria* | Loktanella (g) | AY712185 | 0.966 | Surface water, Sapelo Island, Dean Creek Marsh |
| JML-51 | Eukaryota | *Bacillariophyta* (g) | EF395775 | 0.918 | Surface water, Chesapeake Bay |
| JML-52 | *Alphaproteobacteria* | Rhodobacteraceae (f) | AY568887 | 0.972 | Intertidal flat, Ganghwa Island |
| JML-53 | *Clostridia* | Fusibacter (g) | AB013836 | 0.848 | Deep-sea sediment, Japan Trench |
| JML-54 | *Gammaproteobacteria* | *Gammaproteobacteria* (c) | AY216459 | 0.930 | Temperate estuarine mud |
| JML-55 | *Deltaproteobacteria* | Desulfovibrio (g)^b^ | AJ548465 | 0.621 | Intestinal microflora in *Eriocheir sinensis* |
| JML-56 | Unclassified | *Bacteria* (d) | FJ264777 | 0.695 | Methane seep sediment |
| JML-57 | *Lentisphaerae* | *Lentisphaerae* (c) | EU491113 | 0.870 | Seafloor lavas, Loi'hi Seamount |
| JML-58 | *Flavobacteria* | *Flavobacterium* (g) | EU000234 | 0.984 |  |
| JML-59 | Eukaryota | *Bacillariophyta* (g) | AB426417 | 0.874 | Deep-sea sediment, microbial mat |
| JML-60 | Unclassified | *Bacteria* (d) | FM214821 | 0.579 | Deep-sea sediment, Antarctica |
| JML-61 | *Sphingobacteria* | *Sphingobacteriales* (o) | EU937875 | 0.936 | Riparian iron-oxidizing biofilm |
| JML-62 | *Flavobacteria* | *Algoriphagus* (g) | EF471633 | 0.965 | Surface water, Chesapeake Bay |
| JML-63 | unclassified | *Bacteroidetes* (p) | AM745140 | 0.969 | Marine sediment, Gulf of Mexico |
| JML-64 | *Deltaproteobacteria* | *Desulfobacterium* (g) | AM746260 | 0.654 | Surface sediment, Baltic Sea |
| JML-65 | *Alphaproteobacteria* | *Rhodobacter* (g) | DQ856558 | 0.815 | Intestinal microflora in *Eriocheir sinensis* |
| JML-66 | *Gammaproteobacteria* | *Gammaproteobacteria* (c) | EF459905 | 0.993 | Sediment, Baltic Sea |
| JML-67 | *Deltaproteobacteria* | *Deltaproteobacteria* (c) | FM253592 | 0.917 | Rock biofilm from a gold mine, Poland |
| JML-68 | Unclassified | *Bacteria* (d) | EF602482 | 0.737 | Sediment, Zodletone Spring source , USA |
| JML-69 | *Cyanobacteria* | *GpIIa* (g)^a^ | AY151241 | 1.000 | Subalpine lake |
| JML-70 | *Deltaproteobacteria* | *Deltaproteobacteria* (c) | AM712338 | 0.570 | Biofilm at hydrothermal vent orifice |
| JML-71 | *Bacteroidetes* | *Bacteroidales* (o) | AB121104 | 0.803 | Cold seep sediment, Japan Sea |
| JML-73 | *Anaerolineae* | *Levilinea* (g) | FJ175028 | 0.638 | Rhizosphere in soil |
| JML-74 | *Flavobacteria* | *Winogradskyella* (g) | FJ223290 | 0.901 | Marine sediment/bacterial mat, Antarctica |
| JML-77 | *Cyanobacteria* | *GpIIa* (g)^a^ | AF216952 | 0.991 | Lake Akan, Japan |
| JML-79 | *Cyanobacteria* | GpI (g) ^b^ | AJ293105 | 0.959 | Lake Karpjärvi, Finland |
| JML-80 | *Planctomycetacia* | *Blastopirellula* (g) | EU734947 | 0.953 | Sediment, Bering Sea |
| JML-81 | *Actinobacteria* | *Rubrobacterineae* (so) | EU803238 | 0.882 | Lake Gatun, Panama |
| JML-86 | *Clostridia* | *Clostridiales* (o) | AM501858 | 0.785 | Lagoon sediment, Italy, Venice |
| JML-87 | Unclassified | *Bacteria* (d) | EF061966 | 0.580 | Mangrove sediment, China |
| JML-88 | unclassified | *Firmicutes* (p) | EF445243 | 0.575 | Dairy cow rumen |
| JML-92 | *Gammaproteobacteria* | *Gammaproteobacteria* (c) | AY216459 | 0.923 | Temperate estuarine mud |
| JML-93 | *Gammaproteobacteria* | *Gammaproteobacteria* (c) | AJ876706 | 0.916 | River sediment, Portugal, Alcanena |
| JML-94 | *Cyanobacteria* | *GpIIa* (g)^a^ | AB154316 | 1.000 | Freshwater, Japan, Lake Kasumigaura |
| JML-95 | *Spirochaetes* | *Spirochaeta* (g) | FJ748821 | 0.716 | Sediment, Pearl River estuary |
| JML-96 | *Cyanobacteria* | *GpIIa* (g)^a^ | AB154316 | 1.000 | Freshwater, Lake Kasumigaura |
| Paila10-2 | *Flavobacteria* | *Flavobacteriaceae* (f) | AF273319 | 0.918 | Groundwater |
| Paila10-3 | *unclassified* | *Bacteria* (d) | AB305548 | 0.295 | Hydrothermal sediments |
| Paila10-4 | *Deltaproteobacteria* | *Desulfuromonas* (g) | DQ112371 | 1.000 | Intertidal mudflat sediment |

^a^ Closest isolate hit at NCBI genbank with 98-100% max identity was the genus *Synechococcus.*

^b^ Closest hit was an isolate

**Table S5.** (Continued) Closest hits of RDP sequences (Release 10.10) to the 16S rRNA gene clone sequences from Baltic Sea sediment. GF1 and JML sequences originated from the western Gulf of Finland and P10 from Paimionlahti Bay. Searches were done with the seqmatch tool, version 3 [1,2].

| sequnce code | Class | Lowest rank | Accession number | Score | Isolation source |
| --- | --- | --- | --- | --- | --- |
| Paila10-5 | *Verrucomicrobiae* | *Verrucomicrobiaceae*  *genera incertae sedis (g)* | DQ065053 | 0.845 | Freshwater |
| Paila10-7 | *Nitrospira* | *Magnetobacterium* (g) | AY592165 | 0.800 | Mud volcano sediment |
| Paila10-8 | unclassified | Bacteria (d) | EU918097 | 0.577 | Bahamas stromatolite |
| Paila10-9 | *Deltaproteobacteria* | *Deltaproteobacteria* (c) | EF459870 | 1.000 | Sediment, Baltic Sea |
| Paila10-10 | *Cyanobacteria* | GpIIa (g)^a^ | AJ416277 | 1.000 | Lake water |
| Paila10-11 | unclassified | Bacteria (d) | DQ664146 | 0.594 | Uncontaminated site close to petroleum aquifer |
| Paila10-13 | *Acidobacteria* | Gp6 (g) | FM175349 | 0.879 | Mountain rivulet |
| Paila10-14 | unclassified | Bacteria (d) | AF141567 | 0.825 | Coastal ocean adjacent to Columbia River estuary |
| Paila10-15 | unclassified | *Proteobacteria* (p) | AJ888566 | 0.984 | Lake sediment |
| Paila10-16 | unclassified | Bacteria (d) | AY354165 | 0.853 | Hydrothermal vent sediment |
| Paila10-17 | *Deltaproteobacteria* | *Desulfuromonas* (g) | DQ112371 | 0.951 | Intertidal mudflat sediment |
| Paila10-18 | *Nitrospira* | *Nitrospira* (g) | EU546283 | 0.906 | Lake Washington sediment |
| Paila10-19 | *Gammaproteobacteria* | *Gammaproteobacteria* (c) | EF459868 | 1.000 | Sediment, Baltic Sea |
| Paila10-20 | *Deltaproteobacteria* | *Deltaproteobacteria* (c) | EF459870 | 0.980 | Sediment, Baltic Sea |
| Paila10-21 | unclassified | Bacteria (d) | AB448912 | 0.646 | Deep subseafloor sediments, Gulf of Mexico |
| Paila10-22 | *Deltaproteobacteria* | *Deltaproteobacteria* (c) | EF459870 | 1.000 | Sediment, Baltic Sea |
| Paila10-23 | *Gammaproteobacteria* | *Gammaproteobacteria* (c) | EU546799 | 0.856 | Lake Washington sediment |
| Paila10-24 | *Anaerolineae* | *Anaerolineae* (g) | AM909846 | 0.819 | Rhizosphere soil from rice field |
| Paila10-25 | Eukaryota | *Bacillariophyta* (g) | EF395775 | 0.892 | Surface water, Chesapeake Bay |
| Paila10-27 | *Flavobacteria* | *Flavobacteriaceae* (f) | AF273319 | 0.940 | Groundwater |
| Paila10-28 | *Flavobacteria* | *Flavobacteriaceae* (f) | AF273319 | 0.940 | Groundwater |
| Paila10-29 | unclassified | Bacteria (d) | FJ665192 | 0.822 | Ground swab in mangrove forest |
| Paila10-30 | *Acidobacteria* | Gp7 (g) | AM909905 | 0.630 | Rhizosphere soil from rice field |
| Paila10-31 | unclassified | *Bacteroidetes* (p) | FJ223332 | 0.910 | Marine sediment/bacterial mat, Antarctica |
| Paila10-32 | *Deltaproteobacteria* | *Desulfomonile* (g) | EU595804 | 0.654 | In situ reactor to treat an acid pit mine lake |
| Paila10-33 | unclassified | *Proteobacteria* (p) | EF460067 | 0.751 | Sediment, Baltic Sea |
| Paila10-34 | *Betaproteobacteria* | *Burkholderiales* (o) | DQ829405 | 0.914 | Agricultural soil |
| Paila10-35 | *Cyanobacteria* | GpIIa (g)^a^ | EF395700 | 1.000 | Surface water, Chesapeake Bay |
| Paila10-36 | *Acidobacteria* | Gp6 (g) | EF459910 | 0.918 | Sediment, Baltic Sea |
| Paila10-37 | unclassified | Bacteria (d) | AF141567 | 0.884 | Coastal ocean adjacent to Columbia River estuary |
| Paila10-38 | unclassified | *Proteobacteria* (p) | EF395726 | 0.938 | Surface water, Chesapeake Bay |
| Paila10-39 | *Gammaproteobacteria* | *Gammaproteobacteria* (c) | EF029027 | 0.882 | Reduced sediment associated with *Zostera marina* |
| Paila10-40 | *Spirochaetes* | *Spirochaeta* (g) | EF582502 | 0.811 | Saltmarsh sediment amended with petroleum |
| Paila10-41 | *Acidobacteria* | Gp7 (g) | AM909905 | 0.632 | Rhizosphere soil from rice field |
| Paila10-42 | *Deltaproteobacteria* | Geobacteraceae (f) | EF669171 | 0.602 | Uranium bioremediation site |
| Paila10-43 | *Betaproteobacteria* | Burkholderiales (o) | DQ829405 | 0.917 | Agricultural soil |
| Paila10-44 | unclassified | Bacteria (d) | AF141567 | 0.885 | Coastal ocean adjacent to Columbia River estuary |
| Paila10-45 | *Gammaproteobacteria* | Gammaproteobacteria (c) | EF029027 | 0.882 | Reduced sediment associated with *Zostera marina* |
| Paila10-47 | unclassified | Bacteria (d) | EU284464 | 0.740 | Sediment, Merri Creek |
| Paila10-49 | *Betaproteobacteria* | *Rhodocyclaceae* (f) | AY988609 | 0.965 | Soil |
| Paila10-50 | unclassified | *Bacteroidetes* (p) | FJ223332 | 0.912 | Marine sediment/bacterial mat, Antarctica |
| Paila10-51 | *Deltaproteobacteria* | *Deltaproteobacteria* (c) | EU438360 | 0.800 | Marine sediment |
| Paila10-52 | *Deltaproteobacteria* | Geobacteraceae (f) | EF669171 | 0.604 | Uranium bioremediation site |
| Paila10-53 | *Acidobacteria* | Gp22 (g) | EF459979 | 1.000 | Sediment, Baltic Sea |

^a^ Closest isolate hit at NCBI genbank with 98-100% max identity was the genus *Synechococcus.*

^b^ Closest hit was an isolate

**Table S5.** (Continued) Closest hits of RDP sequences (Release 10.10) to the 16S rRNA gene clone sequences from Baltic Sea sediment. GF1 and JML sequences originated from the western Gulf of Finland and P10 from Paimionlahti Bay. Searches were done with the seqmatch tool, version 3 [1,2].

| Sequnce code | Class | Lowest rank | Accession number | Score | Isolation source |
| --- | --- | --- | --- | --- | --- |
| Paila10-54 | *Nitrospira* | *Magnetobacterium* (g) | EF613377 | 0.562 | In situ reactor in a benzene-contaminated aquifer |
| Paila10-56 | *Anaerolineae* | *Anaerolinea* (g) | FM206155 | 0.832 | Chlorinated ethene-contaminated groundwater |
| Paila10-57 | *Verrucomicrobiae* | *Verrucomicrobiaceae* (f) | DQ444139 | 0.826 | River sediment |
| Paila10-58 | unclassified | Bacteria (d) | EF460096 | 1.000 | Sediment, Baltic Sea |
| Paila10-63 | *Flavobacteria* | *Flavobacteriaceae* (f) | EU542167 | 0.900 | Roots of *Zostera marina* |
| Paila10-64 | *Sphingobacteria* | *Haliscomenobacter* (g) | FM175627 | 0.594 | Mountain rivulet |
| Paila10-65 | *Deltaproteobacteria* | *Desulfuromonaceae* (f) | EF034562 | 0.883 | Permafrost soil |
| Paila10-66 | *Flavobacteria* | *Psychroserpens* (g) | EF215732 | 0.620 | Inert artificial surfaces submerged in |
| Paila10-67 | *WS3** | WS3 genera  incertae sedis (g) | EF395652 | 0.759 | Anoxic bottom water, Chesapeake Bay |
| Paila10-68 | unclassified | Bacteria (d) | AY940555 | 0.688 | Saline Qinghai Lake sediment |
| Paila10-69 | unclassified | Bacteria (d) | FJ197471 | 0.730 | Marine sediment |
| Paila10-70 | *Nitrospira* | *Magnetobacterium* (g) | AY592165 | 0.802 | Mud volcano sediment |
| Paila10-71 | *Nitrospira* | *Magnetobacterium* (g) | AY592165 | 0.802 | Mud volcano sediment |
| Paila10-72 | *Gammaproteobacteria* | *Gammaproteobacteria* (c) | EF459836 | 0.911 | Sediment, Baltic Sea |
| Paila10-73 | *Flavobacteria* | *Psychroserpens* (g) | EF215732 | 0.619 | Artificial surfaces submerged in marine water |
| Paila10-74 | unclassified | Bacteria (d) | AM409925 | 0.839 | Lake profundal sediment |
| Paila10-75 | *Deltaproteobacteria* | *Desulfobacterium* (g) | DQ836867 | 0.900 | Sediment, Baltic Sea |
| Paila10-76 | unclassified | Bacteria (d) | EF392929 | 0.944 | PCB-spiked river sediment |
| Paila10-78 | *Sphingobacteria* | *Crenotrichaceae* (f) | FJ223513 | 0.818 | Marine sediment/ bacterial mat, Antarctica |
| Paila10-79 | unclassified | Bacteria (d) | DQ368325 | 0.740 | Black Sea water |
| Paila10-80 | *Deltaproteobacteria* | *Desulfobulbaceae* (f) | DQ112390 | 0.864 | Intertidal mudflat sediment (saltmarsh) |
| Paila10-81 | *Alphaproteobacteria* | *Sphingopyxis* (g) | AY515408 | 0.966 | German Wadden Sea water |
| Paila10-82 | *Deferribacteres* | *Caldithrix* (g) | EF459950 | 0.978 | Sediment, Baltic Sea |
| Paila10-83 | *Deferribacteres* | *Caldithrix* (g) | EF459950 | 0.978 | Sediment, Baltic Sea |
| Paila10-84 | *Clostridia* | *Ruminococcaceae* *Incertae Sedis* (g) | EU015108 | 0.773 | Membrane bioreactor suspension and biofilm |
| Paila10-85 | *Alphaproteobacteria* | *Roseomonas* (g) | AY509400 | 0.977 | Freshwater bacterioplankton |
| Paila10-86 | unclassified | Bacteria (d) | FM162170 | 0.687 | Reed periphyton |
| Paila10-87 | *Clostridia* | *Clostridia* (c) | FJ037558 | 0.880 | Biofilms in groundwater |
| Paila10-88 | unclassified | Bacteria (d) | AJ786622 | 0.872 | commercial nitrifying inoculum |
| Paila10-89 | *Deltaproteobacteria* | *Deltaproteobacteria* (c) | EF459870 | 0.971 | Sediment, Baltic Sea |
| Paila10-90 | *Cyanobacteria* | GpIIa (g) | EF395708 | 1.000 | Surface water, Chesapeake Bay |
| Paila10-91 | *Deltaproteobacteria* | *Desulfobacterium* (g) | DQ836867 | 0.899 | Sediment, Baltic sea |
| Paila10-92 | *Acidobacteria* | Gp22 (g) | EF459979 | 1.000 | Sediment, Baltic sea |
| Paila10-93 | *Gammaproteobacteria* | *Gammaproteobacteria* (c) | EF459836 | 1.000 | Sediment, Baltic sea |
| Paila10-94 | *Deltaproteobacteria* | Desulfosarcina (g) | EU362222 | 0.919 | Tidal flat sediment |
| Paila10-95 | *Acidobacteria* | Gp23 (g) | AY940556 | 0.867 | Saline lake sediment |
| Paila10-96 | *Alphaproteobacteria* | Sphingopyxis (g) | U63956 | 1.000 | Baltic Sea water |

^a^ Closest isolate hit at NCBI genbank with 98-100% max identity was the genus *Synechococcus.*

^b^ Closest hit was an isolate

**References**

1. Cole JR, Chai B, Farris RJ, Wang Q, Kulam-Syed-Mohideen AS, et al. (2007) The ribosomal database project (RDP-II): introducing *myRDP* space and quality controlled public data. Nucleic Acids Res 35: D169–D172.
2. Cole JR, Wang Q, Cardenas E, Fish J, Chai B, et al. (2009) The Ribosomal Database Project: improved alignments and new tools for rRNA analysis. Nucleic Acids Res 37: D141–D145.
