# Supplementary material for: Phosphorus Chemistry and Bacterial Community Composition Interact in Brackish Sediments Receiving Agricultural Discharges
Source: PLoS One. 2011 Jun 29;6(6):e21555. doi: 10.1371/journal.pone.0021555 (PMC3126828; doi:10.1371/journal.pone.0021555)
Supplement: Dataset S2 — Concentrations of the chemical parameters used in statistical analyses. (DOC) [file pone.0021555.s009.doc]

**Table S2** Concentrations of different chemical forms of phosphorus and elements from phosphorus fractionation extracts (phosphorus-binding elements) as well as total concentrations of elements from sediments that were used in statistical analyses. Data from Lukkari et al. [1-3].

|  |  | Concentrations^a^ | | | | | | | | | | | |
| --- | --- | --- | --- | --- | --- | --- | --- | --- | --- | --- | --- | --- | --- |
|  |  | Reactive P forms | | Immobile P forms | | Elements from P fractionation extracts | | |  | Total elements | | | |
|  | solvent: | NaBD^b^ | NaOH^c^ | NaOH^c^ | HCl^d^ | NaBD^b^ | NaOH^c^ | NaOH^c^ | HCl^d^ |  |  |  |  |
| Sampling station | Depth ^(g)^  (cm) | iP^e^ | NRP^f^ | iP^e^ | iP^e^ | Fe | Si | Al | Mn | Fe | C | N | P |
| Paila10 | 1 | 14.1 | 5.7 | 7.6 | 6.6 | 128.8 | 303.3 | 173.11 | 1.64 | 1139.0 | 2024.4 | 187.8 | 42.2 |
|  | 7 | 12.3 | 5.5 | 10.0 | 6.6 | 64.8 | 269.5 | 170.42 | 1.60 | 1241.0 | 1884.1 | 150.7 | 42.5 |
|  | 15 | 10.6 | 3.3 | 8.4 | 6.1 | 36.5 | 294.1 | 160.63 | 1.43 | 1440.0 | 1722.8 | 125.7 | 36.8 |
|  | 20 | 11.2 | 2.9 | 8.9 | 6.0 | 29.7 | 290.8 | 164.26 | 1.43 | 1062.0 | 1669.0 | 112.3 | 37.9 |
|  | 25 | 11.0 | 2.9 | 8.5 | 6.1 | 50.6 | 303.2 | 172.92 | 1.20 | 1230.0 | 1618.1 | 111.6 | 36.6 |
| Paila14 | 1 | 29.2 | 8.8 | 3.8 | 8.1 | 151.6 | 325.4 | 109.57 | 1.55 | 1393.0 | 2555.0 | 230.5 | 57.6 |
|  | 7 | 25.0 | 6.4 | 8.1 | 7.8 | 55.0 | 339.0 | 133.59 | 1.68 | 1525.0 | 2089.5 | 172.8 | 55.8 |
|  | 15 | 14.6 | 4.3 | 8.6 | 8.1 | 27.0 | 355.9 | 126.21 | 1.91 | 1319.0 | 2072.6 | 170.3 | 44.3 |
|  | 20 | 21.1 | 4.6 | 12.1 | 7.9 | 36.0 | 404.7 | 139.92 | 1.92 | 1210.0 | 2032.3 | 192.4 | 53.9 |
|  | 25 | 13.7 | 4.5 | 8.4 | 7.6 | 30.5 | 430.9 | 150.37 | 1.53 | 1328.0 | 1976.6 | 168.8 | 42.5 |
| AS5 | 1 | 31.8 | 8.8 | 4.2 | 7.9 | 78.8 | 325.1 | 104.00 | 1.87 | 1087.0 | 2762.8 | 355.3 | 60.1 |
|  | 7 | 30.1 | 5.6 | 9.9 | 8.5 | 25.5 | 344.6 | 119.48 | 1.93 | 1439.0 | 2258.5 | 245.9 | 62.1 |
|  | 15 | 19.6 | 5.3 | 8.0 | 8.6 | 26.1 | 403.2 | 128.35 | 1.89 | 1328.0 | 2191.9 | 249.6 | 49.9 |
|  | 20 | 18.6 | 4.8 | 10.3 | 8.4 | 27.0 | 409.5 | 125.09 | 2.04 | 1200.0 | 2103.7 | 254.1 | 51.5 |
|  | 25 | 7.0 | 4.8 | 6.4 | 8.2 | 19.5 | 433.4 | 135.95 | 1.59 | 1034.0 | 2186.6 | 235.7 | 35.2 |
| AS3 | 1 | 21.0 | 10.7 | 3.8 | 10.2 | 145.9 | 361.7 | 122.00 | 1.21 | 947.0 | 2952.3 | 242.8 | 52.2 |
|  | 7 | 7.6 | 7.9 | 3.9 | 11.3 | 24.6 | 363.9 | 91.60 | 1.46 | 983.0 | 2695.1 | 297.1 | 39.8 |
|  | 15 | 4.9 | 7.5 | 4.0 | 10.7 | 21.6 | 368.2 | 105.01 | 1.64 | 1110.0 | 2440.2 | 268.9 | 35.5 |
|  | 20 | 4.9 | 8.7 | 3.3 | 10.2 | 13.4 | 173.1 | 91.80 | 1.47 | 1073.0 | 2300.3 | 259.0 | 35.6 |
|  | 25 | 3.5 | 8.0 | 3.1 | 10.5 | 13.3 | 132.8 | 78.26 | 1.74 | 1075.0 | 2159.2 | 238.4 | 34.5 |
| AS2 | 1 | 29.4 | 20.5 | 5.4 | 10.5 | 63.2 | 562.3 | 64.67 | 1.45 | 796.8 | 5545.5 | 640.1 | 77.4 |
|  | 7 | 4.0 | 13.8 | 3.2 | 11.8 | 20.2 | 429.5 | 53.24 | 2.93 | 964.6 | 4104.4 | 484.0 | 43.3 |
|  | 15 | 4.0 | 14.0 | 3.0 | 10.8 | 19.3 | 297.1 | 59.13 | 2.19 | 1007.6 | 3880.6 | 425.8 | 42.8 |
|  | 20 | 3.8 | 13.2 | 3.1 | 11.5 | 14.5 | 221.3 | 54.17 | 2.59 | 951.9 | 3727.4 | 418.0 | 42.3 |
|  | 25 | 3.8 | 13.1 | 3.2 | 11.0 | 12.5 | 244.0 | 55.61 | 2.64 | 996.9 | 3570.2 | 387.3 | 42.1 |
| AS7 | 1 | 2.5 | 7.6 | 1.7 | 12.8 | 21.2 | 327.7 | 33.71 | 1.15 | 833.0 | 4047.9 | 417.4 | 31.5 |
|  | 15 | 1.1 | 4.9 | 1.4 | 13.5 | 2.6 | 222.7 | 34.97 | 1.75 | 900.0 | 2100.0 | 171.8 | 28.9 |
|  | 20 | 1.0 | 4.2 | 1.3 | 14.4 | 1.3 | 192.8 | 35.72 | 1.64 | 887.3 | 1729.0 | 144.4 | 28.8 |
|  | 25 | 1.1 | 3.3 | 1.2 | 14.4 | 2.0 | 161.4 | 33.95 | 1.63 | 1035.9 | 1468.0 | 120.0 | 26.0 |
| JML | 1 | 4.0 | 11.2 | 1.5 | 6.4 | 18.9 | 313.8 | 23.91 | 0.87 | 635.0 | 6347.8 | 580.2 | 37.0 |
|  | 7 | 1.7 | 8.9 | 2.3 | 13.4 | 10.3 | 528.5 | 50.65 | 1.75 | 844.3 | 3893.9 | 372.5 | 35.8 |
|  | 15 | 1.4 | 4.9 | 1.6 | 12.8 | 3.0 | 180.8 | 37.47 | 1.60 | 839.5 | 2500.5 | 200.2 | 28.2 |
|  | 25 | 2.2 | 4.7 | 0.8 | 12.8 | 32.6 | 210.4 | 35.28 | 2.10 | 965.5 | 2030.0 | 172.4 | 26.7 |
| GF1 | 1 | 7.3 | 14.0 | 2.1 | 10.4 | 63.5 | 341.1 | 26.22 | 1.41 | 691.2 | 7431.6 | 795.1 | 47.5 |
|  | 7 | 2.1 | 7.4 | 1.6 | 13.9 | 55.7 | 461.8 | 41.57 | 2.56 | 871.9 | 2551.5 | 271.0 | 32.9 |
|  | 15 | 1.9 | 4.3 | 1.6 | 13.4 | 6.4 | 317.0 | 37.63 | 2.17 | 727.4 | 2319.3 | 242.8 | 27.1 |
|  | 20 | 1.3 | 2.5 | 0.9 | 11.3 | 10.4 | 132.8 | 27.46 | 1.40 | 648.0 | 1507.3 | 146.3 | 19.9 |
|  | 25 | 1.4 | 3.0 | 0.6 | 13.2 | 6.3 | 137.0 | 24.45 | 2.87 | 701.0 | 1630.0 | 161.4 | 23.6 |
| C63 | 1 | 13.4 | 10.7 | 2.0 | 13.4 | 26.9 | 361.9 | 39.05 | 0.75 | 566.6 | 3821.5 | 428.4 | 46.2 |
|  | 2 | 2.6 | 11.5 | 2.2 | 12.4 | 15.0 | 473.6 | 42.33 | 0.70 | 643.6 | 3859.0 | 428.4 | 34.4 |
|  | 5 | 2.5 | 10.6 | 2.3 | 11.7 | 42.1 | 724.7 | 51.21 | 1.19 | 636.6 | 3729.9 | 414.1 | 33.0 |
|  | 10 | 2.5 | 6.7 | 1.4 | 10.8 | 19.4 | 334.0 | 41.26 | 1.26 | 605.4 | 2356.2 | 278.4 | 27.0 |

^a^ µmol g^-1^ dry weight (DW)

^b^ Sodium dithionite in sodium bicarbonate

^c^ Sodium hydroxide

^d^ Hydrochloric acid

^e^ Inorganic phosphorus

^f^ Labile organic phosphorus

^g^ Depth from sediment-water interface.

**References:**

1. Lukkari K, Leivuori M, Hartikainen H (2008) Vertical distribution and chemical character of sediment phosphorus in two shallow estuaries in the Baltic Sea. Biogeochemistry 90: 171–191.
2. Lukkari K, Leivuori M, Vallius H, Kotilainen A (2009a) The chemical character and burial of phosphorus in shallow coastal sediments in the northeastern Baltic Sea. Biogeochemistry 94: 141–162.
3. Lukkari K, Leivuori M, Kotilainen A (2009b) Trends in chemical character and burial of sediment phosphorus from open sea to organic rich inner bay in the Baltic Sea. Biogeochemistry 96: 25-48.
